# Onco-immunomodulatory properties of pharmacological interference with RAS-RAF-MEK-ERK pathway hyperactivation

**DOI:** 10.3389/fonc.2022.931774

**Published:** 2022-07-27

**Authors:** Thomas Yul Avery, Natalie Köhler, Robert Zeiser, Tilman Brummer, Dietrich Alexander Ruess

**Affiliations:** ^1^ Department of General and Visceral Surgery, Center of Surgery, Medical Center University of Freiburg, Freiburg, Germany; ^2^ Department of Medicine I - Medical Center, Medical Center University of Freiburg, Freiburg, Germany; ^3^ CIBSS - Centre for Integrative Biological Signalling Studies, University of Freiburg, Freiburg, Germany; ^4^ German Cancer Consortium Deutsches Konsortium Translationale Krebsforschung (DKTK), partner site Freiburg, German Cancer Research Center Deutsches Krebsforschungszentrum (DKFZ), Heidelberg, Germany; ^5^ Institute of Molecular Medicine and Cell Research (IMMZ), Faculty of Medicine, University of Freiburg, Freiburg, Germany; ^6^ Comprehensive Cancer Center Freiburg (CCCF), Faculty of Medicine, Medical Center University of Freiburg, Freiburg, Germany

**Keywords:** MAPK signaling, KRAS/BRAF mutations, targeted inhibition, tumor immunity, immune escape, immunomodulation, immunotherapy

## Abstract

Hyperactivation of the RAS-RAF-MEK-ERK cascade - a mitogen-activated protein kinase pathway – has a well-known association with oncogenesis of leading tumor entities, including non-small cell lung cancer, colorectal carcinoma, pancreatic ductal adenocarcinoma, and malignant melanoma. Increasing evidence shows that genetic alterations leading to RAS-RAF-MEK-ERK pathway hyperactivation mediate contact- and soluble-dependent crosstalk between tumor, tumor microenvironment (TME) and the immune system resulting in immune escape mechanisms and establishment of a tumor-sustaining environment. Consequently, pharmacological interruption of this pathway not only leads to tumor-cell intrinsic disruptive effects but also modification of the TME and anti-tumor immunomodulation. At the same time, the importance of ERK signaling in immune cell physiology and potentiation of anti-tumor immune responses through ERK signaling inhibition within immune cell subsets has received growing appreciation. Specifically, a strong case was made for targeted MEK inhibition due to promising associated immune cell intrinsic modulatory effects. However, the successful transition of therapeutic agents interrupting RAS-RAF-MEK-ERK hyperactivation is still being hampered by significant limitations regarding durable efficacy, therapy resistance and toxicity. We here collate and summarize the multifaceted role of RAS-RAF-MEK-ERK signaling in physiology and oncoimmunology and outline the rationale and concepts for exploitation of immunomodulatory properties of RAS-RAF-MEK-ERK inhibition while accentuating the role of MEK inhibition in combinatorial and intermittent anticancer therapy. Furthermore, we point out the extensive scientific efforts dedicated to overcoming the challenges encountered during the clinical transition of various therapeutic agents in the search for the most effective and safe patient- and tumor-tailored treatment approach.

## Introduction

As stated in the World Health Organization’s (WHO) Global Cancer Observatory (GLOBOCAN) report of 2021, cancer incidence and mortality are rapidly growing on a global scale, forming an increasing health burden and important barrier to increasing life expectancy (1). As reported in the WHO report on Global Health Estimates 2020, cancer has become the first or second leading cause of death before the age of 70 years in 112 of 183 countries, surpassing mortality rates of stroke and coronary heart disease in many countries (2). Estimating future development based on growth and aging of the population, worldwide annual new cancer cases are projected to grow 47% in the year 2040 to an estimated 28.4 million cases per year compared to 19.3 million cases in 2020 (1). This projection will very likely be an underestimation of the true development in the light of increasing prevalence of cancer-associated risk factors in many countries of the world (1). Therefore, it comes as no surprise that a tremendous amount of scientific effort is being devoted to cancer research in order to increase our knowledge in the areas of prevention, screening, diagnosis, effective interventions, and surveillance and to aid in their tailored integration into national health care plans to reduce the future burden and suffering from cancer.

In an attempt to understand the mechanistic background of cancer on a molecular basis, scientific investigations in the recent decades have led to the discovery of the causal relationship between the pathologically hyperactivated RAS-RAF-MEK-ERK signaling cascade – a mitogen-activated protein kinase (MAPK) pathway – and development and progression of various leading tumor entities, including non-small cell lung cancer (NSCLC), colorectal carcinoma (CRC), pancreatic ductal adenocarcinoma (PDAC), and malignant melanoma (3). Elements of this signaling cascade have been identified as potential oncogenes, ultimately leading to pathway hyperactivation and promotion of proliferative and tumorigenic signals (3). Of the associated oncogenes, mutant Kirsten rat sarcoma viral oncogene homolog (KRAS) and BRAF have been identified as the major driving forces behind the RAS-RAF-MEK-ERK pathway hyperactivation. Mutant KRAS appears to be involved in over 90% of PDAC, about 50% of CRC, about 30% of NSCLC and to a lesser extent in other tumors, while mutant BRAF has primarily been observed in melanomas (3–5). Consequently, the RAS-RAF-MEK-ERK pathway and its associated regulatory feedback loops as well as upstream activators and downstream effector proteins have been brought into the focus of the search for potential therapeutic targets in the attempt to slow down, put a halt to, or even reverse the oncogene-driven process (3). These efforts have led to the identification of promising targets for pharmacological inhibition and interruption of RAS-RAF-MEK-ERK hyperactivation. Furthermore, an increasing body of evidence shows that oncogenic mutations are capable of mediating autocrine and paracrine crosstalk between tumor cells, the tumor microenvironment (TME) and various subsets of immune cells in order to establish and maintain a pro-tumorigenic environment and employ effective immune evasion mechanisms at different stages of the so-called cancer-immunity cycle (6, 7).

Selective pharmacologic targeting of elements of the RAS-RAF-MEK-ERK signaling cascade has led to encouraging results in both preclinical and clinical studies through direct inhibition of oncogenic signaling (8). Importantly, a closer look at the role of the immune system in tumor growth and its influence on the establishment and maintenance of the TME revealed that the systemically administered targeted therapies are capable of achieving multifaceted anti-tumorigenic immunomodulation (9–15). Within this context, especially MEK inhibitors (MEKi) have received a substantial amount of scientific attention due to their preclinically observed potential to significantly alter the tumor-associated pro-tumorigenic immune response towards an anti-tumorigenic inflammatory response (10, 13). However, preclinical studies have not uniformly shown consistency in the aforementioned beneficial immunomodulatory effects of MEK inhibition. Furthermore, transition to the clinical setting has been hampered due to significant toxicity, occurrence of resistance mechanisms and unequivocal clinical results (**Table 1**) (10, 23–26). Nevertheless, pharmacological interruption of ERK signaling is being investigated extensively in both the preclinical and clinical setting, especially as part of various combinatorial and/or intermittently administered therapy regimens (**Table 2**) (8). Furthermore, the combination of immune checkpoint blockade (ICB) and RAS-RAF-MEK-ERK signaling inhibition has been identified as a promising approach in tumors with high immunogenicity (9, 10, 27). However, further research elucidating the immunomodulatory anti-tumor effects and associated consequences for the complex interactions in the TME is still necessary.

**Table 1:** Results from selected completed clinical trials investigating MEK inhibition as part of dual and triple therapy regimens in patients with KRAS-/BRAF-mutant solid tumors and melanoma.

Author (year) (reference)	Study Type	Study Design	Outcome	
Ascierto et al. (2019) (16)	Phase II dbRCT	Dabrafenib/trametinib/pembrolizumab vs. dabrafenib/trametinib/placebo in patients with BRAF-mutant melanoma	*Median PFS*	16 months vs. 10 months
			*12-month PFS*	59.3% vs. 45.2%
			*12-month OS*	79% vs. 72%
			*ORR*	63% vs. 51% (complete response 18% vs. 12%)
			*TRAE*	Manageable with dose reduction, interruption or discontinuation
Hellmann et al. (2019) (17)	Phase I/II	Combinatorial cobimetinib/atezolizumab therapy in patients with solid tumors including mCRC, melanoma, and NSCLC	*12-month PFS*	11% (mCRC), 50% (melanoma), 29% (NSCLC)
			*OS*	43% (mCRC), 85% (melanoma), 57% (NSCLC)
			*Prevalence of KRAS/BRAF mutations*	68% (KRAS, mCRC), 46% (BRAF, melanoma), 43% (KRAS, NSCLC)
			*ORR (mut vs. wt)*	9% vs. 8% (mCRC), 40% vs. 50% (melanoma), 8% vs. 33% (NSCLC)
			*TRAE*	Manageable with dose reduction or interruption
			*IHC Analysis*	↑CD8+ T cell infiltrates and upregulation of MHC class I molecules
Ribas et al. (2019) (18)	Phase I/II	Concomitant administration of dabrafenib/trametinib/pembrolizumab in patients with BRAF-mutant metastatic melanoma	*Median PFS*	15.4 months
			*ORR*	73% of participants with complete or partial response
			*TRAE*	Manageable with dose reduction and corticosteroid treatment
			*IHC Analysis, RNAseq*	↑MHC class I/II expression, ↑CD8+ T cells, upregulated genes involved in CD8+ T cell function
Sullivan et al. (2019) (19)	Phase Ib	Atezolizumab/cobimetinib/vemurafenib in 4 cohorts with various lead-in periods in BRAF-mutated melanoma patients	*Chosen regimen*	Cohort 4, 28-day lead-in period with cobimetinib and vemurafenib before commencing with atezolizumab
			*Median PFS*	12.9 months
			*Estimated 2-year OS*	~75%
			*ORR*	71.8%, with all patients showing tumor reduction in target lesions and complete response in 20% of patients
			*TRAE*	Manageable, discontinuation in 28.2% of patients
			*IHC Analysis*	↑CD8+ T cell infiltration
Gutzmer et al. (2020) (20)	Phase III dbRCT	Atezolizumab/cobimetinib/vemurafenib vs. placebo/cobimetinib/vemurafenib in patients with untreated BRAF-mutant melanoma	*Median PFS*	15.1 months vs. 10.6 months
			*TRAE*	Manageable, 13% vs. 16% of patients stopped all treatment due to AE
Yarchoan et al. (2021) (21)	Phase II open-label RCT	Cobimetinib vs. cobimetinib/atezolizumab in advanced unresectable biliary tract cancer	*Median PFS*	1.87 months vs. 3.65 months
			*12-month PFS*	0% vs. 13%
			*OS*	No significant effect
			*ORR*	No significant effect
			*TRAE*	Manageable with dose reduction and treatment interruptions
			IHC Analysis	↑Ratio of CD8+ T/FoxP3+ Treg in biopsies
Zimmer et al. (2021) (22)	Phase I/II	Encorafenib/binimetinib/pembrolizumab in patients with BRAF-mutant melanoma	*12-month PFS*	41%
			*ORR*	64%
			TRAE	Manageable

AE, adverse events, IHC, immunohistochemistry, mCRC, metastatic colorectal carcinoma, mut, mutant, NSCLC, non-small cell lung cancer, ORR, overall response rate, OS, overall survival, PFS, progression-free survival, RCT, randomized controlled trial, RNAseq, RNA sequencing data, TRAE, treatment-related adverse effects, wt, wild-type.

**Table 2:** Overview of currently active clinical trials investigating various combinations of MEK inhibition, other small molecule inhibitors and immunotherapy in mutant KRAS- and BRAF-driven malignancies.

NCT Number	Title	Status	Conditions	Interventions	Phases
NCT02224781	Dabrafenib and Trametinib Followed by Ipilimumab and Nivolumab or Ipilimumab and Nivolumab Followed by Dabrafenib and Trametinib in Treating Patients with Stage III-IV BRAFV600 Melanoma	Active, not recruiting	Melanoma	Drug: Dabrafenib, Ipilimumab, Nivolumab, Trametinib	Phase III
NCT02631447	Sequential Combo Immuno and Target Therapy (SECOMBIT) Study	Active, not recruiting	Melanoma	Drug: LGX818, MEK162, Nivolumab, Ipilimumab	Phase II
NCT02858921	Neoadjuvant Dabrafenib, Trametinib and/or Pembrolizumab in BRAF Mutant Resectable Stage III Melanoma	Active, not recruiting	Melanoma	Drug: Dabrafenib, Trametinib, Pembrolizumab	Phase II
NCT03149029	Abbreviated MAPK Targeted Therapy Plus Pembrolizumab in Melanoma	Active, not recruiting	Melanoma	Drug: Pembrolizumab, Dabrafenib,Trametinib	Phase II
NCT02625337	Study Comparing Pembrolizumab with Dual MAPK Pathway Inhibition Plus Pembrolizumab in Melanoma Patients	Unknown	Melanoma	Drug: Pembrolizumab, Dabrafenib, Trametinib	Phase II
NCT02130466	A Study of the Safety and Efficacy of Pembrolizumab (MK-3475) in Combination with Trametinib and Dabrafenib in Participants with Advanced Melanoma (MK-3475-022/KEYNOTE-022)	Completed	Melanoma, solid tumors	Drug: Pembrolizumab, Dabrafenib, Trametinib, Placebo	Phase I/II
NCT02902042	Encorafenib + Binimetinib + Pembrolizumab in Patients with Unresectable or Metastatic BRAF V600 Mutant Melanoma (IMMU-TARGET)	Completed	Melanoma	Drug: Encorafenib, Binimetinib, Pembrolizumab	Phase I/II
NCT03299088	Pembrolizumab and Trametinib in Treating Patients with Stage IV Non-Small Cell Lung Cancer and KRAS Gene Mutations	Active, not recruiting	NSCLC	Drug: Pembrolizumab, Trametinib	Phase I
NCT03989115	Dose-Escalation/Expansion of RMC-4630 and Cobimetinib in Relapsed/Refractory Solid Tumors and RMC-4630 and Osimertinib in EGFR Positive Locally Advanced/Metastatic NSCLC	Active, not recruiting	Solid tumor	Drug: RMC-4630, Cobimetinib, Osimertinib	Phase I/II
NCT04916236	Combination Therapy of RMC-4630 and LY3214996 in Metastatic KRAS Mutant Cancers	Recruiting	Pancreatic cancer, CRC, NSCLC, KRAS-mutation related tumors	Drug: RMC-4630, LY3214996	Phase I
NCT05195632	Combination of Encorafenib and Binimetinib in BRAF V600E Mutated Chinese Patients With Metastatic Non-Small Cell Lung Cancer	Recruiting	NSCLC	Drug: Encorafenib, Binimetinib	Phase II
NCT04967079	MEK Inhibitor Combined With Anlotinib in the Treatment of KRAS-mutated Advanced Nonsmall Cell Lung Cancer	Recruiting	NSCLC	Drug: Trametinib, Anlotinib	Phase I
NCT04965818	Phase 1b/2 Study of Futibatinib in Combination With Binimetinib in Patients With Advanced KRAS Mutant Cancer	Recruiting	Advanced or metastatic solid tumors, NSCLC	Drug: Futibatinib, Binimetinib	Phase I/II
NCT04720417	Defactinib and VS-6766 for the Treatment of Patients With Metastatic Uveal Melanoma	Recruiting	Metastatic uveal melanoma	Drug: Defactinib, Raf/MEK inhibitor VS-6766	Phase II
NCT04739566	Dabrafenib and Trametinib Combination as a Neoadjuvant Strategy in BRAF-positive Anaplastic Thyroid Cancer	Recruiting	Thyroid Gland Anaplastic Carcinoma	Drug: Dabrafenib, Trametinib	Phase II
NCT04720768	Encorafenib, Binimetinib and Palbociclib in BRAF mutant Metastatic Melanoma CELEBRATE	Recruiting	Melanoma, metastasis	Drug: Binimetinib, Encorafenib, Palbociclib	Phase I/II
NCT04675710	Pembrolizumab, Dabrafenib, and Trametinib Before Surgery for the Treatment of BRAF Mutated Anaplastic Thyroid Cancer	Recruiting	Thyroid Gland Anaplastic Carcinoma, Thyroid Gland Squamous Cell Carcinoma	Drug: Dabrafenib, Pembrolizumab, Trametinib Intervention: Intensity-modulated radiation therapy	Phase II
NCT04625270	A Study of VS-6766 v. VS-6766 + Defactinib in Recurrent Low- Grade Serous Ovarian Cancer With and Without a KRAS Mutation	Recruiting	Ovarian Cancer	Drug: VS-6766 and Defactinib	Phase II
NCT04620330	A Study of VS-6766 v. VS-6766 + Defactinib in Recurrent G12V, Other KRAS and BRAF Non-Small Cell Lung Cancer	Recruiting	NSCLC	Drug: VS-6766 and Defactinib	Phase II
NCT04566133	Combination of Trametinib (MEK Inhibitor) and Hydroxychloroquine (HCQ) (Autophagy Inhibitor) in Patients With KRAS Mutation Refractory Bile Tract Carcinoma (BTC).	Recruiting	Bile Duct Cancer, Biliary Cancer, Biliary Tract Neoplasms, Cholangiocarcinoma	Drug: Trametinib, Hydroxychloroquine	Phase II
NCT04543188	A FIH Study of PF-07284890 in Participants With BRAF V600 Mutant Solid Tumors With and Without Brain Involvement	Recruiting	Malignant melanoma, NSCLC	Drug: PF-07284890, Binimetinib, Midazolam	Phase I
NCT04526782	ENCOrafenib With Binimetinib in bRAF NSCLC	Recruiting	NSCLC	Drug: Encorafenib, Binimetinib	Phase II
NCT04418167	JSI-1187-01 Monotherapy and in Combination With Dabrafenib for Advanced Solid Tumors With MAPK Pathway Mutations	Recruiting	Solid tumors	Drug: JSI-1187, Dabrafenib	Phase II
NCT04310397	Dabrafenib, Trametinib, and Spartalizumab for the Treatment of BRAF V600E or V600K Mutation Positive Stage IIIB/C/D Melanoma	Recruiting	Melanoma	Drug: Dabrafenib, Spartalizumab, Trametinib Procedure: Therapeutic Conventional Surgery	Phase 2
NCT04214418	Study of Combination Therapy With the MEK Inhibitor, Cobimetinib, Immune Checkpoint Blockade, Atezolizumab, and the AUTOphagy Inhibitor, Hydroxychloroquine in KRASmutated Advanced Malignancies	Recruiting	Gastrointestinal Cancer	Drug: Cobimetinib, Hydroxychloroquine, Atezolizumab	Phase I/II
NCT03981614	Binimetinib and Palbociclib or TAS-102 in Treating Patients With KRAS and NRAS Mutant Metastatic or Unresectable Colorectal Cancer	Recruiting	Metastatic Colorectal Carcinoma	Drug: Binimetinib, Palbociclib, Trifluridine and Tipiracil Hydrochloride	Phase II
NCT03975231	Dabrafenib, Trametinib, and IMRT in Treating Patients With BRAF Mutated Anaplastic Thyroid Cancer	Recruiting	Thyroid Gland Anaplastic Carcinoma	Drug: Dabrafenib, Trametinib Radiation: Intensity-Modulated Radiation Therapy	Phase I
NCT03905148	Study of the Safety and Pharmacokinetics of BGB-283 (Lifirafenib) and PD-0325901 (Mirdametinib) in Participants With Advanced or Refractory Solid Tumors	Recruiting	Solid tumor	Drug: Lifirafenib, Mirdametinib	Phase I/II
NCT03875820	Phase I Trial of Defatcinib and VS-6766.	Recruiting	NSCLC, low grade serous ovarian cancer, endometrioid carcinoma, pancreatic cancer	Drug: VS-6766, Defactinib	Phase I
NCT03839342	Binimetinib and Encorafenib for the Treatment of Advanced Solid Tumors With Non-V600E BRAF Mutations	Recruiting	Solid tumor	Drug: Binimetinib, Encorafenib	Phase II
NCT03754179	Dabrafenib/Trametinib/Hydroxychloroquine for Advanced Pretreated BRAF V600 Mutant Melanoma	Recruiting	Melanoma	Drug: Dabrafenib, Trametinib, Hydroxychloroquine	Phase I/II
NCT03554083	NeoACTIVATE: Neoadjuvant Therapy for Patients With High Risk Stage III Melanoma	Recruiting	Melanoma	Drug: Atezolizumab, Cobimetinib, Vemurafenib, Tiragolumab	Phase II
NCT03543969	Adaptive BRAF-MEK Inhibitor Therapy for Advanced BRAF Mutant Melanoma	Recruiting	Melanoma	Drug: Vemurafenib, Cobimetinib	Phase I
NCT03430947	Vemurafenib Plus Cobimetinib After Radiosurgery in Patients With BRAF-mutant Melanoma Brain Metastases	Recruiting	Melanoma	Drug: Vemurafenib, Cobimetinib	Phase II
NCT03244956	Efficacy of MEK (Trametinib) and BRAFV600E (Dabrafenib) Inhibitors With Radioactive Iodine (RAI) for the Treatment of Refractory Metastatic Differentiated Thyroid Cancer	Active, not recruiting	Metastatic Radioactive Iodine Refractory Thyroid Cancer Patients With RAS or BRAF Mutation	Drug: Trametinib, Dabrafenib, rhTSH Radiation: 131I	Phase I
NCT03225664	Trametinib and Pembrolizumab in Treating Patients With Recurrent Non-small Cell Lung Cancer That Is Metastatic, Unresectable, or Locally Advanced	Active, not recruiting	Metastatic NSCLC, recurrent NSCLC, unresectable NSCLC	Drug: Trametinib, Pembrolizumab	Phase I/II
NCT03175432	Bevacizumab and Atezolizumab With or Without Cobimetinib in Treating Patients With Untreated Melanoma Brain Metastases	Recruiting	Advanced melanoma	Drug: Atezolizumab, Bevacizumab, Cobimetinib	Phase II
NCT03170206	Study of the CDK4/6 Inhibitor Palbociclib (PD-0332991) in Combination With the MEK Inhibitor Binimetinib (MEK162) for Patients With Advanced KRAS Mutant Non-Small Cell Lung Cancer	Recruiting	Lung cancer	Drug: Binimetinib, Palbociclib	Phase I/II
NCT03101254	LY3022855 With BRAF/MEK Inhibition in Patients With Melanoma	Active, not recruiting	Melanoma	Drug: LY3022855, Vemurafenib, Cobimetinib	Phase I/II
NCT03065387	Neratinib and Everolimus, Palbociclib, or Trametinib in Treating Participants With Refractory and Advanced or Metastatic Solid Tumors With EGFR Mutation/Amplification, HER2 Mutation/Amplification, or HER3/4 Mutation or KRAS Mutation	Recruiting	Advanced malignant solid neoplasm	Drug: Everolimus, Neratinib, Palbociclib, Trametinib	Phase I
NCT02645149	Molecular Profiling and Matched Targeted Therapy for Patients With Metastatic Melanoma	Recruiting	Melanoma	Drug: Trametinib and/or supportive care, CDK4/6 and MEK inhibitor	Phase II
NCT02642042	Trametinib and Docetaxel in Treating Patients With Recurrent or Stage IV KRAS Mutation Positive Non-small Cell Lung Cancer	Active, not recruiting	Advanced NSCLC	Drug: Docetaxel, Trametinib	Phase II
NCT02231775	Dabrafenib and Trametinib Before and After Surgery in Treating Patients With Stage IIIB-C Melanoma With BRAF V600 Mutation	Recruiting	Melanoma	Drug: Dabrafenib, Trametinib Procedure: Therapeutic Conventional Surgery	Phase II
NCT02079740	Trametinib and Navitoclax in Treating Patients With Advanced or Metastatic Solid Tumors	Recruiting	Metastatic/refractory/Unresectable malignant solid neoplasm	Drug: Trametinib, Nacitoclax	Phase I/II
NCT02022982	PALBOCICLIB + PD-0325901 for NSCLC & Solid Tumors	Active, not recruiting	NSCLC, solid tumors	Drug: Palbociclib, PD-0325901	Phase I/II
NCT01933932	Assess Efficacy & Safety of Selumetinib in Combination With Docetaxel in Patients Receiving 2nd Line Treatment for v-Kiras2 Kirsten Rat Sarcoma Viral Oncogene Homolog (KRAS) Positive NSCLC	Active, not recruiting	Advanced or metastatic NSCLC	Drug: Selumetinib, Docetaxel, Pegylated GCSF	Phase III
NCT01909453	Study Comparing Combination of LGX818 Plus MEK162 Versus Vemurafenib and LGX818 Monotherapy in BRAF Mutant Melanoma	Active, not recruiting	Melanoma	Drug: LGX818, MEK162, Vemurafenib	Phase III
NCT01859026	A Phase I/IB Trial of MEK162 in Combination With Erlotinib in NSCLC Harboring KRAS or EGFR Mutation	Active, not recruiting	Lung cancer, NSCLC	Drug: MEK162, Erlotinib	Phase I
NCT01682083	Dabrafenib With Trametinib in the Adjuvant Treatment of High-risk BRAF V600 Mutationpositive Melanoma (COMBI-AD).	Active, not recruiting	Melanoma	Drug: Dabrafenib, Trametinib	Phase III

https://clinicaltrials.gov. CRC, Colorectal cancer, NSCLC, Non-small cell lung cancer.

We here collate and summarize the properties of the RAS-RAF-MEK-ERK signaling pathway from physiology to oncoimmunology, highlighting its function in physiologic cell signaling, its implications in tumor development, tumor maintenance and immune-evasion, but also its importance in homeostasis of immune cells. We further outline the rationale and concepts for exploitation of immunomodulatory properties of RAS-RAF-MEK-ERK inhibition with a focus on novel therapeutic agents and therapy regimens. In this update, we discuss relevant results from selected recent (pre-)clinical studies and in particular the anti-tumor and immunomodulatory effects associated with MEK inhibition to point out its potential in the context of combinatorial and intermittent anticancer therapy.

## The RAS-RAF-MEK-ERK signaling cascade in physiology and immune function

The intracellular RAS-RAF-MEK-ERK signaling cascade is classified as a MAPK pathway, a group of signaling pathways each consisting of three distinct cytosolic protein kinase components, that form a functional signaling module serving the purpose of relaying extracellular signals to the cell nucleus in order to alter the expression pattern of genes promoting proliferation and/or differentiation. In an upstream-to-downstream fashion, the three protein kinase groups are collectively called MAP kinase kinase kinases (MAPKKK), MAP kinase kinases (MAPKK), and MAP kinases (MAPK), also known as the classical three-tiered MAPKKK-MAPKK-MAP kinase cascade. There are several kinases that belong to each of the kinase groups and form a functional unit or signaling pathway with each of their upstream and downstream counterparts (28). The MAP kinases can be classified into conventional and atypical enzymes dependent on the ability of MAPKK members to phosphorylate and activate them, as reviewed extensively elsewhere (29, 30). Conventional MAP kinases are substrates of the MAPKK family and as such are regulated by the classical three-tiered MAPKKK-MAPKK-MAP kinase cascade, whereas the regulation and physiological function of atypical MAP kinases is much more complex and still unclear (29). There are four important members belonging to the group of conventional MAP kinases, i.e. the extracellular signal-regulated protein kinases (ERK1/2), ERK5, the p38 MAP kinases, and the c-Jun NH_2_-terminal kinases (JNK) (28). Atypical MAP kinases are ERK3/4, the Nemo-like kinase (NLK), and ERK7/8 (29). Despite the similarities in structure and regulation, in particular at the level of MAPKK and MAPKs, the various typical MAPK pathways fulfil partially overlapping but also unique functions, such as promoting proliferation and differentiation in case of ERK, while relaying stress and pro-apoptotic signals in case of the p38 kinases. *In vitro* many MAPK share several substrates, but to what extent the various MAPK can complement each other in these phosphorylation events in living cells remains an area of investigation. Lastly, it should be kept in mind that in mammalian cells almost every MAPK pathway-signaling element is represented by various isoforms as a result of gene duplication and splice variants, thereby contributing to the fine tuning of signaling intensities but also complicating pharmacological intervention.

Focusing on the RAS-RAF-MEK-ERK pathway, the ERK1/2 MAP kinases are effector proteins of the MAPKKs MEK1/2 (28). The MAPKKs themselves are activated by different MAPKKKs, most importantly the group of RAF kinases, which are in turn activated by various upstream signals (31). In order to activate the MAPK pathways, an extracellular signal has to be first recognized by the cell through the means of various receptors on its membrane surface, including transmembrane receptor tyrosine kinases (RTKs), cytokine receptors, integrin receptors, G-protein coupled receptors (GPCRs) and TGFβ-receptors. These receptors then transmit the signal to the intracellular space by either phosphorylation or binding and therefore activation of specific kinases. This step leads to the formation of an intracellular signaling complex that can relay the signal onward. Major effector proteins of RTKs and activators of the RAF-MEK-ERK pathway are proteins belonging to the RAS family, which consists of monomeric guanosine triphosphate (GTP)-loaded GTPases capable of activating various different signaling pathways besides the RAF-MEK-ERK pathway, e.g. the PI3K-AKT pathway and the Ral Guanine Nucleotide Dissociation Stimulator (RalGDS) pathway (32, 33). After RAF-MEK-ERK activation, pERK1/2 phosphorylates numerous cytoplasmic substrates, but also translocates into the cell nucleus where a large spectrum of substrates and transcription factors are phosphorylated and activated leading to increased transcriptional activity of genes associated with cell metabolism, cell cycle progression, survival, and differentiation (34). Additionally, the seemingly linear cascade of the MAPK pathway is regulated by complex crosstalk and regulatory feedback loops at every level, which together lead to a finely tuned homeostasis as comprehensively reviewed elsewhere (3, 35). Regarding functional consequences of the activation of the different members of the RAS-RAF-MEK-ERK pathway, studies using genetically engineered mouse models have revealed that the three isoforms of the RAF protein, i.e., RAF1 (c-RAF), ARAF, and BRAF, are all capable of activating MEK1/2 by phosphorylation (3, 36). Yet, differences in the functional consequence dependent on the active RAF protein isoform exist for embryonic development. In mice, deletion of *Raf1* led to increased levels of apoptosis in several tissues and defects in vascularization and placental development (37, 38). Deletion of *Braf* on the other hand led to general growth retardation and vascular defects due to endothelial cell apoptosis (39). *Araf*-deficient mice showed a wide range of phenotypic expression ranging from minor neurological abnormalities with long-term survival to more severe neurological and intestinal defects and postnatal death (3). It is noteworthy, that systemic ablation of *Raf1* or *Braf* in adult mice, alone or in combination, did not lead to significant toxicities (40). However, in the case of ablation of all three RAF isoforms, cell proliferation ceased and death followed (41). Lastly, it needs to be kept in mind that the MAPKKK and proto-oncogene product TPL2/COT confer RAF independent activation downstream of Toll-like receptors in inflammatory settings (42). Regarding the function of MEK1/2, deletion of *Mek2* in mice showed dispensability for embryonic development and adult homeostasis (3). However, *Mek1-*deficient mice died during embryonic development due to placental defects. Deletion of both genes also led to embryonic death (43). Systemic ablation of *Mek1* and *Mek2* in adult mice caused rapid death due to severe intestinal defects (40). Similar results were found in studies regarding ERK-deficient mice, showing dispensability of *Erk1* and embryonic lethality in case of *Erk2* disruption (44). Taken together, the mentioned studies revealed significant dependency of embryonic development and adult homeostasis on all three main nodes of the RAF-MEK-ERK pathway (3).

ERK signaling regulates many physiologic cellular processes and responses in a wide variety of immune cells (45). Here, we focus specifically on T and B lymphocytes, NK cells, NKT cells, macrophages, DCs, and neutrophils, since these immune cell types have been demonstrated to take on important roles in the interaction with tumor cells and the associated TME tilting the balance either towards a pro- or anti-tumor setting. Since selective pharmacologic targeting of elements of the MAPK pathway has shown promise in the battle against cancer, it is of great interest to identify possible implications of MAPK and especially RAS-RAF-MEK-ERK signaling interference in specific immune cell types.

### T lymphocytes

**Figure 1:**
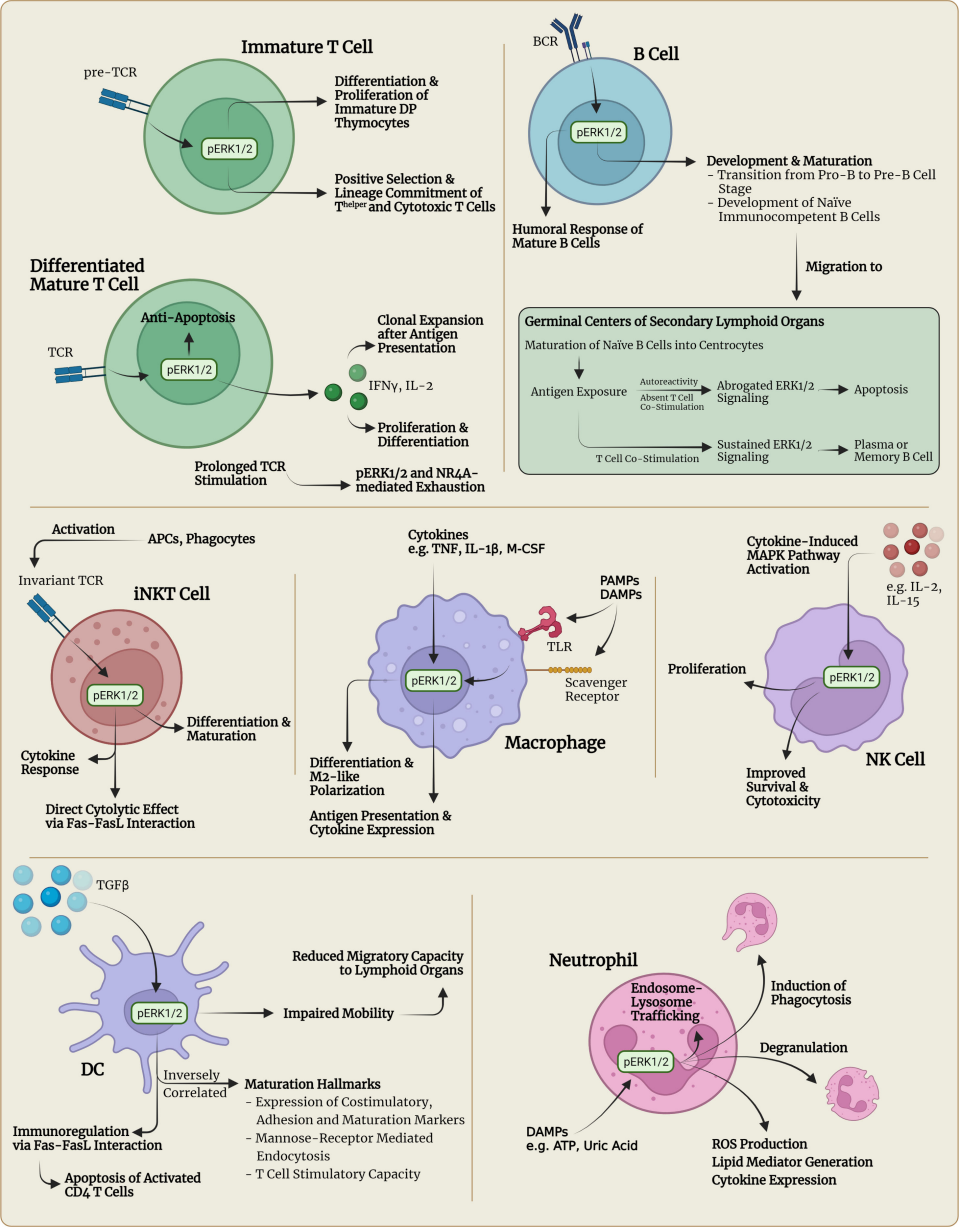
Visualization of selected immunophysiological consequences of pERK1/2 signaling in different immune cell types. APC, antigen-presenting cell; ATP, adenosine triphosphate; BCR, B cell receptor, DAMPs, damage-associated molecular patterns; DC, dendritic cell; DP thymocytes, double positive (CD4^+^CD8^+^) thymocytes; IL, interleukin; IFN, interferon; M-CSF, macrophage colony-stimulating factor; NK, natural killer; NKT, natural killer T; PAMPs, pathogen-associated molecular patterns; ROS, reactive oxygen species; TCR, T cell receptor; TLR, Toll-like receptor; TNF, tumor-necrosis factor.

In T lymphocytes, distinctive mechanisms activate the three classical three-tiered MAPK pathways described earlier. For example, the activation of the T cell antigen receptor (TCR) can lead to ERK activation, whereas co-stimulation by accessory molecules of the TCR, e.g. CD28, can lead to JNK activation and therefore transcription of different subsets of genes (28). The MAPK pathways are already involved in early thymocyte development when thymocytes go through the stages of double negative (CD4^-^CD8^-^) (DN) thymocytes, double positive (CD4^+^CD8^+^) (DP) thymocytes and finally maturation into either CD4^+^CD8^-^ T^helper^ cells or CD4^-^CD8^+^ T^cytotoxic^ cells (**Figure 1**). While the p38 MAPK pathway is involved in the progression from DN to DP thymocytes, the ERK pathway seems to be crucial for differentiation and proliferation of immature DP thymocytes after activation of the pre-TCR. Also, the ERK pathway was shown to be involved in the positive selection and lineage commitment of future T^helper^ or T^cytotoxic^ cells (**Figure 1**) (28). Interestingly, various studies have shown that pharmacological inhibition of the ERK pathway seems to skew thymocyte lineage commitment towards CD8^+^ T^cytotoxic^ cells, mediated by the kinase ERK2 (46, 47). The JNK pathway on the other hand was suggested to be involved in the negative selection (apoptosis) of DP thymocytes with insufficient affinity of the pre-TCR to the corresponding major histocompatibility complex (MHC) molecule or autoreactivity to self-antigens (28). After positive selection and lineage commitment, differentiation of CD4^+^ T^helper^ cells into T^helper^ 1 (Th1) or T^helper^ 2 (Th2) cells does not seem to be solely dependent on the presence of specific transcription factors involved in cytokine gene regulation, e.g., T-bet or GATA3, but seems to be regulated by activation of MAPK pathways. It was shown, that the ERK1/2 signaling pathway is required for the differentiation of CD4^+^ T cells into Th2 cells through stabilization of GATA3, whereas intact JNK and p38 signaling appeared to be a requirement for interferon (IFN)γ production and subsequent differentiation of CD4^+^ T cells into Th1 cells (28, 48). How the MAPK signaling cascade determines developmental and functional aspects of other T^helper^ subsets, including Th9, Th17, Th22 or T follicular helper (Tfh) cells has not yet been completely clarified and requires more research. In effector CD8^+^ T^cytotoxic^ cells, the cytotoxic activity seems to be regulated by the ERK pathway, as studies on pharmacologic ERK inhibition or in *Erk* deficient mouse models have suggested (28, 47). The role of the JNK pathway in CD8^+^ T cells is unclear, whereas p38 signaling was demonstrated to lead to the induction of apoptosis in CD8^+^ T cells but not in CD4^+^ T cells by decreased expression of the anti-apoptotic protein Bcl-2 (49). Furthermore, intact ERK signaling was shown to be required for the regulation of the expression and release of cytokines such as interleukin-2 (IL-2), a crucial cytokine for the proliferation and differentiation of T cells and priming with subsequent proliferation and survival of naïve T cells in response to antigen presentation (**Figure 1**) (11, 50, 51). Interestingly, it was discovered that specific loss or inhibition of Erk2 leads to a severe defect in IL-2 production accompanied by decreased expansion and survival of *Erk2^T-/-^* CD8^+^ T cells *in vitro* and *in vivo* of a murine model (47). Exogenous IL-2 could only partially rescue the Erk2-deficient CD8^+^ T cells. IL-2 was also shown to be critical for regulatory T cell function, which promote an immunosuppressive tumor microenvironment (52). Activation and functional capacity remained intact in Erk2-deficient CD8^+^ T cells, as well as priming and early expansion in response to antigen presentation (47). In contrast, absence of Erk1 was found to be largely dispensable for CD8^+^ T cell proliferation and survival (47). Further investigation into the regulation of survival and apoptosis in *Erk2^T-/-^* CD8^+^ T cells showed that upregulation of pro-survival regulators and downregulation of pro-apoptotic regulators are Erk2 dependent (**Figure 1**) (47). The Erk2-dependency of survival and apoptosis seems particularly relevant in T cell response contraction after activation. In this context, Programmed cell death protein 1 (PD-1) and cytotoxic T-lymphocyte-associated protein 4 (CTLA-4) signaling have been identified to exert immunoregulatory functions, i.e., suppression of T cell activation, reduction of proinflammatory cytokine expression and induction of apoptosis, in response to TCR-mediated activation and proliferation (10).

### B lymphocytes

In B lymphocytes, intact RAS-RAF-MEK-ERK signaling has been implicated in several developmental stages such as maturation, selection, expansion, differentiation and survival (53). For instance, the RAS-RAF-MEK-ERK pathway has been found to be crucial in successful cell cycle progression in the process of B cell development, particularly regarding the pre-B cell receptor driven transition from the pro-B to the pre-B cell stage (**Figure 1**) (54, 55). Pre-BCR but also B cell antigen receptor (BCR) signaling has been shown to activate three distinct protein tyrosine kinases, LYN, SYK and BTK, which are capable of activating various signaling pathways including the RAS-RAF-MEK-ERK pathway (56, 57). Once pre-B cells continue development and reach the stage of naïve immunocompetent B cells, they undergo a complex priming and maturation process within microanatomic structures located in secondary lymphoid organs called germinal centers (GC) (**Figure 1**). In the event of BCR stimulation in combination with costimulatory signals from T^helper^ cells, naïve B cells transform into centroblasts that undergo clonal expansion and somatic hypermutation (58, 59). Subsequently, centroblasts differentiate into centrocytes and migrate towards the light zone of a GC where the centrocytes are confronted with follicular dendritic cells (FDCs) that display unprocessed antigen on their surface (59). In case the centrocytes express BCRs with high affinity to the presented antigen, they take up the antigen from FDCs, internalize and process it so that it can be presented to T cells. If these T cells recognize the presented antigen, they provide costimulatory signaling including CD40-ligation to induce survival and further differentiation of centrocytes into antibody secreting plasma cells or memory B cells. Autoreactive centrocytes on the other hand do not receive costimulatory T cell signaling and therefore go into BCR-triggered apoptosis (**Figure 1**) (59). Investigations into the mechanisms of selection have revealed that the outcome of the underlying BCR signaling has a biphasic course of action (60). In the first 12h after antigen presentation, BCR signaling activates ERK1/2 (early signaling) with subsequent phosphorylation of prosurvival Bcl-2 family proteins and proapoptotic Bim. The phosphorylation of Bcl-2 proteins was shown to lead to a delay in BCR-induced apoptosis, whereas phosphorylation of Bim inhibited its proapoptotic activity by preventing its association with Bax, a proapoptotic protein (60–62). The resulting anti-apoptotic signaling then gives GC B cells time to process and present antigenic protein structures to T^helper^ cells. Once the GC B cells receive T^helper^-mediated CD40L-CD40 costimulatory signals, the RAF-MEK-ERK-mediated anti-apoptotic signaling is sustained and the selected B cells can differentiate into memory B cells or plasma cells (60). Indeed, ablation of ERK1/2 in GC B cells was shown to significantly compromise plasma cell differentiation (63). However, if no costimulatory signal is received after antigenic presentation to T^helper^ cells, ERK1/2 activity undergoes BCR-induced inhibition (late signaling) and as a result the B cells will die within the next 12 h through apoptosis induced by downregulation of Bcl-2 proteins and accumulation of Bim (**Figure 1**) (60). Interestingly, intact KRAS-signaling was shown to play a significant role in mediating BCR-induced RAF-MEK-ERK signaling and consequent cell proliferation and survival in the development and differentiation of mature B cells in a murine model, as KRAS deficiency led to a marked reduction of mature B cells and impairment of cell proliferation and survival (64). However, the *in vivo* B cell humoral response was only minimally affected by KRAS deficiency, suggesting that the remaining ERK1/2 activity in the absence of KRAS is sufficient for mounting a B-cell immune response with comparable antibody production levels as seen in control conditions (64). Concluding, it has become clear that RAF-MEK-ERK signaling is crucial in key developmental stages in the production of immunocompetent, functional B lymphocytes but not in mounting a humoral response. Also, RAF-MEK-ERK signaling is not just an all-or-nothing phenomenon regarding cell survival or apoptosis, but can show contextual ambiguity. To which degree the RAF-MEK-ERK signaling cascade is involved in other areas of B lymphocyte physiology is still being actively researched today. A very recent study investigated the role of BRAF and RAF1 in murine B cell development. Although both BRAF and RAF1 are expressed in B cells and are both activated by BCR stimulation, their requirement for B cell development and function appears context dependent as the conditional knock-out of both isoforms mainly affected the transition of pro-B cells into pre-B cells and the differentiation of activated B cells into plasma cells (65). This study, however, did not functionally address to which extent ARAF and the enigmatic KSR pseudokinases could potentially compensate for the loss of BRAF and RAF in circumstances in which these two isoforms were found to be less relevant. Nevertheless, BRAF plays an important role in some B cell neoplasms as virtually 100% of typical hairy cell leukemia and about 1 to 5% of chronic lymphocytic leukemia and myeloma contain *BRAF* mutations (66). As recently reviewed elsewhere, the impact of the neoantigen-driven humoral response of B lymphocytes on tumorigenesis is increasingly being acknowledged and valued in understanding the complex interactions of the immune system with the TME (67).

### NK cells

As comprehensively reviewed elsewhere, natural killer (NK) cells are a subset of the heterogeneous group of innate lymphoid cells (ILC) that develop from common lymphoid progenitor cells but do not carry a genetically rearranged antigen receptor (68). Besides their role in the protection against pathogens, NK cells are known to be capable of carrying out receptor-mediated anti-tumor cell cytotoxicity and specifically modulating the DC and T lymphocyte immune response in a contact- and cytokine/chemokine-dependent way (68). Similar to T lymphocytes, the RAS-RAF-MEK-ERK signaling cascade has been implicated to be a crucial signaling node in the process of proliferation, survival and cytotoxicity of NK cells (**Figure 1**) (69–71). In this context, especially IL-15, the most important cytokine for NK cell development, and IL-2 were shown to be capable of activating downstream signaling pathways including the RAS-MEK-ERK, JAK-STAT5 and PI3K/AKT pathways to sustain cell expansion and cytolytic function (72, 73).

### NKT cells

Natural killer T (NKT) cells are developmentally related to conventional T lymphocytes and carry surface markers characteristic of both NK cells and memory T lymphocytes – hence the label NKT cells (74, 75). Importantly, type 1 NKT (iNKT) cells express an invariant TCR capable of recognizing lipid-presenting CD1 molecules – mainly expressed by professional APCs and phagocytes - and carry important functions in both innate and adaptive immunity regulation by rapidly secreting large amounts of cytokines upon TCR stimulation and bridging both types of immune responses, reviewed comprehensively elsewhere (**Figure 1**) (74, 76). Furthermore, NKT cells were implied to play an important role in tumor regression through cytokine-mediated NK or cytolytic T cell activation, stimulation of pro-inflammatory IL-12 production by DCs and subsequent enhanced T cell proliferation, and direct cytolytic effects through Fas-FasL interaction (**Figure 1**) (74). Regarding the impact of the RAF-MEK-ERK signaling cascade on NKT cells, previous studies discovered a large dependency of NKT cell differentiation and maturation on the RAS-MEK-ERK pathway, similar to the development and differentiation of conventional T lymphocytes as described above (77). However, intact RAS-MEK-ERK signaling did not appear to be important in NKT cell survival, but was rather dependent on the mammalian target of rapamycin (mTOR) signaling pathway (78). In inflammatory responses, previous studies identified a crucial role of the RAS-RAF-MEK-ERK and JNK pathways in mounting the pivotal cytokine response of NKT cells responsible for their multifaceted effector functions (78).

### Macrophages

Macrophages are phagocytic cells that act on various pathogen- and/or damage-associated molecular patterns (PAMPs, DAMPs) detected by specific pattern recognition receptors such as Toll-like receptors (TLRs), scavenger receptors and integrins (79–81). The recognition of such PAMPs and DAMPs, the presence of proinflammatory cytokines, e.g. tumor necrosis factor (TNF) and IL-1β, and physical-chemical changes in the extracellular milieu caused by environmental stress all lead to the initiation of signaling pathways cumulating in the activation of NF-κB and MAP kinases (**Figure 1**). The resulting production of proinflammatory and regulatory cytokines and internalization and degradation of recognized PAMPs and/or DAMPs leads to enhanced antigen presentation to CD4^+^ T^helper^ and invariant NKT cells (81, 82). The development of macrophages is dependent on the presence of the cytokine M-CSF, which has been shown to activate the ERK1/2 pathway, amongst others, driving macrophage growth and development (81). Further investigations into the importance of intact ERK1/2 signaling have revealed a critical dependence specifically in the terminal stages of macrophage development from monocytes, as ablation of ERK1/2 was shown to lead to a defective cell proliferation and differentiation in said late stage development of macrophages but not in myeloid precursor cells or monocytes (81). The physiological role of BRAF in the monocytic-macrophage/dendritic cell lineage remains ill-defined, but somatic BRAF and MEK mutations are increasingly found in histiocytic neoplasms and represent a target for inhibitors blocking these kinases (83).

### Dendritic cells

DCs act as the sentinels of the immune system, continuously scanning their environment for pathogens and foreign antigens. Once a pathogen or foreign antigen is encountered, the DCs are activated and migrate to secondary lymphoid tissues in order to execute their function as antigen-presenting cells (APCs). DCs are potent APCs responsible for the initiation of primary immune responses by interacting with naïve CD4^+^ T^helper^ cells and leading to CD8^+^ T cell activation *via* cross-presentation. Furthermore, they contribute to the induction and maintenance of immunological tolerance through various mechanisms such as immunosuppressive action as well as induction of apoptosis of activated effector T cells (84, 85). It has been proposed that different subsets of DCs at different developmental and functional stages could explain the large variety of DC functions, as described elsewhere (86). Interestingly, recent research efforts have revealed the existence of DCs with immunoregulatory capacities that express TGF β-induced and ERK1/2-mediated high levels of Fas ligand (FasL) and are capable of inducing apoptosis of activated CD4^+^ T cells *via* Fas-FasL interaction in order to negatively regulate T cell responses and maintain immune homeostasis (**Figure 1**) (86). In regard to the maturation process of DCs, activity of the RAF-MEK-ERK signaling pathway was found to be inversely correlated with maturation hallmarks including expression of costimulatory, adhesion and maturation surface proteins, loss of mannose-receptor-mediated endocytosis, and IL-12 and T-lymphocyte stimulatory capacity (**Figure 1**). Activity levels of the p38 MAPK pathway on the other hand were shown to be positively correlated with the overall maturation process (87). Also, ERK1/2 activity seems to be correlated with impaired mobility of DCs, inhibiting migratory capacity to egress from tissues and migrate to lymphoid tissues (88).

### Neutrophils

As comprehensively reviewed elsewhere (89, 90), neutrophils play a central role in the inflammatory response of innate immunity as professional phagocytes and source of various cytokines and chemokines influencing initiation, orchestration and maintenance of adaptive immune responses. Regarding the importance of the RAF-MEK-ERK pathway and other MAPK pathways for neutrophil physiology, previous studies have indicated that the ERK1/2 and p38 MAPK signaling pathways are involved in key physiologic cellular processes such as reactive oxygen species (ROS) production, lipid mediator generation, cytokine production, the process of degranulation, and regulation of chemotaxis (**Figure 1**) (91–93). Furthermore, it was shown that traumatic injury and tissue necrosis initiate a systemic inflammatory response syndrome (SIRS) through the release of damage associated molecular patterns (DAMPs), such as ATP (94) or uric acid (**Figure 1**) (95), which are readily recognized by circulating neutrophils and activate the ERK1/2 and p38 pathways leading to the mounting of a pro-inflammatory response (96). Also, neutrophil extracellular traps (NETs) - decondensed nuclear chromatin associated with proteins and released by neutrophils during an inflammatory response in order to prevent pathogen dissemination and exert immunomodulatory effects – were shown to activate effector functions of neutrophils through the activation of ERK1/2, p38 and AKT signaling cascades (97). In contrast, the JNK pathway was shown to take on a less pivotal role in neutrophil function than the ERK1/2 and p38 pathways (98). Intact JNK signaling was implicated in controlling delayed apoptosis in neutrophils (98). Furthermore, RAF-MEK-ERK signaling in concert with PI3K/AKT signaling was shown to regulate endosome-lysosome trafficking and the induction of phagocytosis, which is critical for inflammatory resolution (99). Surprisingly, it was shown in a neutrophil-specific fashion, that MEK and ERK can be activated independently of each other dependent on the extracellular stimulatory signal, resulting in either complementary or redundant effects, e.g., cytokine generation and delayed apoptosis, respectively (93). Under discrete stimulatory conditions, ERK1/2 is activated by a TGFβ-activated kinase 1 (TAK1)-controlled signaling cascade rather than the MAPK pathway and appears to be a pivotal signaling molecule regulating cytokine transcription (93). This phenomenon should be taken into account in inflammatory responses consisting of a strong neutrophilic component and might warrant the utilization of concurrent ERK inhibition, especially when investigating therapeutic strategies involving MEK inhibition (93).

Taken together, MAPK- in general and RAS-RAF-MEK-ERK-signaling in particular bears important implications in immune cell physiology from development to activation and homeostasis. The resulting consequences for targeted inhibition in an oncologic context will be discussed later on.

## The RAS-RAF-MEK-ERK signaling cascade in oncogene-driven tumorigenesis and maintenance

Hyperactivation of various elements of the RAS-RAF-MEK-ERK pathway is known to play a crucial role in a large variety of tumors. The respective pathologically altered genes have been recognized as oncogenes (3). The most frequently observed mutations occur in KRAS, an isoform of the RAS family that code for various families of monomeric GTPases. Once activated by binding to GTP, the GTPases transmit intracellular signals and catalyze the inactivation of the bound GTP molecule to GDP. Most of these mutations are missense mutations of the codon 12, corresponding to glycine (G), which lead to changes from glycine to another amino acid, most frequently aspartic acid (D), valin (V), or cysteine (C) (33, 100). Oncogenic mutant RAS proteins can have varying degrees of intrinsic catalytic ability dependent on specific mutations and consequently remain GTP-bound and activated for varying durations (32, 100, 101). Furthermore, it has also been shown that mutant RAS is capable of increased self-activation and therefore decreased dependence on its associated regulatory proteins by spontaneously exchanging GDP for GTP (101). Also, mutant RAS isoforms can be partially or even completely insensitive to associated regulatory GTPase activating proteins (GAPs), such as neurofibromin (NF1) or p120GAP, and guanine nucleotide exchange factors (GEFs), such as Son of Sevenless homolog 1/2 (SOS1/2) and Ras guanyl nucleotide-releasing protein (RasGRP) (100). The occurrence of these phenomena and the associated frequency auf auto-activation are highly dependent on the specific mutation of the RAS protein, as extensively discussed elsewhere (101). Other mutated isoforms of RAS, namely the H- and N-isoforms, have also been implicated in human cancer, e.g. melanoma, bladder cancer, and acute myeloid leukemia, however to a lower extent in comparison to KRAS (5).

Other mutations frequently observed in human cancers regard the *RAF* isoform *BRAF*. In >90% of cases, the substitution of valine 600 by a glutamic acid residue (V600E) leads to a constitutively active kinase independent of upstream RAS signaling, while other *BRAF* mutations still require post-translational dimerization but also function independent of upstream signaling (102). In mechanistic murine studies on RAS-driven tumors, *Braf* and *Raf1* have been discovered to be either crucial or completely dispensable in the initiation of tumor development dependent on disease entity (3, 40, 103–106). Ablation of *Raf1* induced significant tumor regression, including a complete disappearance in 10% of tumors, likely as a consequence of increased rates of apoptosis (3). Various studies have shown that Raf1 is capable of phosphorylating proteins outside of the canonical MAPK pathway, e.g., RB1, MST2, ASK IκB, and Bad, implying the ability of Raf1 to influence pro- and anti-apoptotic processes and to control cellular migration and adhesion *via* ROKα (107–111).

Less frequent mutations within the RAF-MEK-ERK cascade occur in the genes *ARAF, MEK1, MEK2* and *ERK1/2*. Although these genes have been reported to function as oncogenes, they are either rarely found in human tumors or their implication in tumorigenesis has yet to be defined more clearly (112). Mutations regarding regulatory proteins of RAS have also been implicated in enhancing tumorigenesis as well as development of therapy resistance and include *NF1*. *NF1* is frequently co-mutated with codon 13 mutations of *KRAS* and *PTPN11*. *PTPN11* encodes the Src homology region 2 domain-containing phosphatase-2 (SHP2) and can lead to so-called RASopathies, i.e., clinically defined genetic syndromes caused by germline mutations of regulators or components of the MAPK pathway (100).

In a physiologic state, the activation of RAS triggered by activated RTKs is usually short-lived. Tyrosine-specific protein phosphatases reverse the phosphorylation which contributed to the activation of RAS. The nature of an extracellular signaling molecule, e.g., EGF or NGF, influences the subsequent duration and extent of the MAPK pathway activation by modulating the intracellular signaling complex and influencing the response of positive and negative feedback loops (35). Depending on this modulation, the cellular response can vary greatly from induced proliferation to cessation of proliferation and a switch to differentiation. The presence of built-in negative feedback loops on many different levels however makes sure that, once triggered, the MAP kinase module is shut off at a given time to restore homeostasis. In oncogene-driven tumorigenesis, the regulatory feedback mechanisms are rendered ineffective by perpetuated hyperactivation of the MAPK pathway (3). Different pathologically altered mechanisms can lead to distorted intracellular signaling culminating in the aforementioned hyperactivation including continuous expression of various oncogenes such as RTK mutations and amplification of wild-type genes (3, 113).

### Targeting RAS

Since the most frequently observed mutation leading to RAS-RAF-MEK-ERK pathway hyperactivation regards mutant KRAS, the KRAS oncoproteins have been the focus of a tremendous amount of research efforts in the search of therapeutic interventions targeting KRAS. However, efforts to develop selective inhibitors of KRAS oncoproteins have historically been hampered due to its small molecule size, relatively smooth and shallow surface lacking apparent binding sites for small molecules, interaction with multiple associated regulatory proteins and its picomolar affinity for GTP/GDP. Thus, potential candidates targeting RAS oncoproteins – alone or in combination regimens - proved to be either clinically insignificant or too toxic in early-phase clinical trials. The successful development of KRAS^G12C^ specific small molecule inhibitors covalently binding to the codon 12 cysteine residue therefore implicates nothing less than a paradigm shift (33). Promising results in phase I/II clinical trials in patients suffering from KRAS-driven NSCLC evaluating sotorasib, a highly selective and irreversible inhibitor leading to trapping of KRAS in an inactive GDP-bound state, have led to a recent approval by the Food and Drug Administration (FDA) (114–119). In the phase II trial by Skoulidis et al., the objective response rate to sotorasib was 37.1%, the median progression-free survival 6.8 months and the median overall survival 12.5 months with comparably low toxicity, providing supportive evidence of the importance and potential of sotorasib in the treatment of patients with pretreated advanced KRAS^G12C^-mutated NSCLC (119). A recent trial evaluating another KRAS^G12C^ inhibitor adagrasib by Jänne et al. uncovered similar results in patients with pretreated advanced NSCLC, paving the way for another impending medical approval (120). However, the most commonly observed KRAS mutations in PDAC and CRC, KRAS^G12D^ and KRAS^G12V^, are not targetable with cysteine-directed molecules like sotorasib (33). Interestingly, a noncovalent, potent KRAS^G12D^ inhibitor was recently discovered through extensive structure-based drug design with promising and robust anti-tumor efficacy in a murine model awaiting further substantiation (121). However, even though KRAS^G12C^ inhibitors have shown promising activity in patients harboring the KRAS^G12C^ mutation, progression of the disease nevertheless occurs due to mechanisms of acquired resistance as a consequence of secondary KRAS mutations or acquired bypass mechanisms on multiple levels, e.g. activating mutations in NRAS or BRAF, oncogenic fusions involving ALK or RAF1, or epithelial-mesenchymal-transition (EMT) and dedifferentiation (33). In continuation of drug discovery, several preclinical studies on selective, direct-acting covalent inhibitors of KRAS^G12C^ are currently active with no efficacy results reported yet (33). Furthermore, KRAS-directed therapeutic approaches based on novel mechanisms such as vaccines, adoptive T cell therapy, proteolysis-targeting chimeras (PROTACs), and CRISPR/Cas9 technology are emerging and are being currently investigated (33).

### Targeting RAF, MEK1/2, and ERK1/2

The first important drug capable of inhibiting RAF-signaling was sorafenib, a multi-kinase inhibitor, which was ultimately approved for treatment of kidney and liver tumors (122). In the following years, inhibitors with higher specificity toward specific mutant RAF isoforms were developed, e.g. vemurafenib, dabrafenib, or encorafenib, which target and inhibit mutant BRAF^V600E/V600K^ proteins in melanoma (123). However, these RAF inhibitors were found to induce paradoxical ERK activation through a mechanism in which active (not necessarily mutant) RAS promotes the formation of BRAF homo- or heterodimers in which the drug-bound protomer induces allosteric transactivation of the drug-free partner and subsequent MEK/ERK activation (102, 124). This paradoxical ERK activation promoted the development of squamous skin cancer in patients carrying latent RAS mutations in skin cells. Furthermore, these RAF inhibitors were shown to promote the development of secondary neoplasms such as various types of leukemia and solid tumors including melanoma and PDAC as reported in vemurafenib-treated patients (125, 126). In an effort to prevent such paradoxical inhibitor action, RAF inhibitors known as paradox breakers were developed, e.g. PLX4032, which are capable of disrupting BRAF-containing dimers (127, 128). Other efforts to develop specific RAF inhibitors, in particular RAF dimer inhibitors, have not yet yielded significant results or are limited by toxicity and are still in early phase clinical trials (3). In KRAS-driven lung cancer, targeting of RAF1 was suggested to be a tumor-selective strategy since RAF1 was found to be essential for tumor initiation (40, 129, 130). In a murine model, systemic ablation of *Raf1* did not seem to cause significant toxicities, which is a major advantage compared to MEK or ERK inhibitors (131). However, RAF1 inhibition requires high specificity since interference with other RAF isoforms could lead to significant toxicity, as studies on panRAF kinase inhibitors have shown (132). Attempts to target RAF1 more specifically have led to the discovery of therapeutics interfering with the interaction of RAF1 and its selective pro-apoptotic effectors, e.g. ROK α, ASK1, and MST2 (133). Furthermore, pharmacological, proteasome-mediated selective degradation of RAF1 by means of proteolysis-targeting chimeras (PROTAC) could lead to another strategy in specifically targeting RAF1 (134). To date, however, RAF1 degradation by PROTACs has noy yet been successfully tested (135, 136).

Important druggable targets in the RAS-RAF-MEK-ERK pathway were found to be MEK1/2, which can be considered central components of this signaling pathway. Of the MEKi that have been developed so far, some have already been approved for the treatment of melanoma, e.g. trametinib, in combination with selective BRAF^V600E^ inhibitors, e.g. dabrafenib (137). In other cancer entities, therapy regimens with MEKi as monotherapy or in combination with chemotherapy have failed to demonstrate significant survival benefits (138–140). Possibly, administration of adequate dosages for a sufficient anti-tumor response was limited by the occurrence of significant toxicities at higher dosages, e.g. anemia, thrombocytopenia, febrile neutropenia, transaminase elevation, and uveitis besides more common side effects including skin toxicity, diarrhea and fatigue (3, 32). Also, inhibition of one effector pathway might result in compensatory activation of parallel survival and proliferation pathways, e.g., the PI3K-AKT pathway and STAT3 activation (32, 140, 141). Other concerning observations were either (i) the presence of intrinsic resistance of tumor cells mediated by for example a feedback loop that activates EGFR signaling and induces cell survival/proliferation, (ii) the rapid development of acquired resistance to the administered MEKi through various mechanisms, including insufficient inhibition of ERK activity, loss of feedback-inhibition, (re-)activation or upregulation of diverse RTKs such as MET, IGF-1R, EGFR or HER2 leading to cell growth and proliferation, newly acquired mutations in MEK1/2 or the emergence of other oncoproteins, such as RAS/RAF amplification or mutation, that eventually lead to ERK1/2 reactivation, and (iii) the evolution of cross-resistance between targeted MAPK pathway inhibition and immunotherapy in sequential combinatorial therapeutic approaches (123, 141–147).

Particularly fueled by reactivation of ERK1/2 after previous treatment with MEKi, ERK kinase inhibitors (ERKi) were developed, e.g. ulixertinib and ONC201, with the intention of overcoming MEKi-associated limitations. ERKi are currently being evaluated in various phase I/II clinical trials either as monotherapy or in combination with chemotherapy, i.e., paclitaxel and gemcitabine (32, 123, 132). Similar to MEKi, ERKi have been shown to cause significant toxicities when administered in high dosages. Studies are currently investigating the possibility of selectively inhibiting key substrates further downstream of ERK1/2 with the hope of causing less toxicities (3).

Nevertheless, targeted therapies and immunotherapies have revolutionized the treatment of patients with metastatic cancer with durable tumor control in specific patient subsets warranting further investigation and refinement of these therapeutic modalities, also to increase understanding of resistance mechanisms and how to avoid or circumvent these mechanisms (3, 8).

### Targeting RAS regulatory proteins

In recent years, regulatory proteins of RAS activity have been identified as eligible drug targets including the SHP2 and Son of SOS1/2. SHP2 is a non-receptor protein tyrosine phosphatase (PTP) encoded by *PTPN11* that is involved in relaying signals downstream of several growth factor, cytokine and integrin RTKs (148). The activity of SHP2 has been shown to be required for full activation of the RAS-MAPK pathway. Furthermore, SHP2 has a role as a negative regulator of the JAK-STAT signaling cascade (148–150). The involvement and requirement of SHP2 in these signaling pathways has uncovered its essential role in oncogenic signaling, for example linked to PDAC and NSCLC (151). Extensive research efforts have demonstrated that SHP2 seems to be required for proper function of mutant KRAS, prominently during carcinogenesis (151). Loss or inhibition of SHP2 in established tumors was shown to slow down tumor progression. Furthermore, once downstream RAS effector proteins are pharmacologically inhibited, intact SHP2 signaling appeared be required for the reestablishment of RAS signaling through a multiple RTK-dependent feedback reactivation mechanism, e.g., EGFR, HER and FGFR signaling (152). Therefore, one could argue that intact SHP2 signaling plays an important role in keeping up intrinsic and acquired resistance mechanisms to circumvent pharmacologic inhibition of the RAF-MEK-ERK signaling cascade. Indeed, Fedele et al. and Ryan et al. found indications supporting this notion as the development of adaptive resistance to prolonged MEK inhibition or KRAS^G12C^ inhibition in a murine model could be prevented by co-administration of a SHP2 inhibitor (152, 153). Taken together, these findings warrant further investigation in the human setting. Currently, multiple clinical trials are actively recruiting participants to this end, combining targeted inhibition of MEK or ERK with SHP2 inhibition (e.g., NCT03989115, NCT04916236) (**Table 2**).

In the recent years, fragment-based and high-throughput screening approaches have led to the discovery of small molecules capable of disrupting the KRAS-SOS1 interaction (154). Interestingly, some of these small molecules were shown to activate rather than inhibit the SOS1-mediated nucleotide exchange leading to a biphasic modulation of RAS signaling through negative feedback on SOS1 (154, 155). As an example, a recent study demonstrated that selective inhibition of SOS1 with a nanomolar inhibitor effectively downregulated levels of active RAS in tumor cells (154). Importantly, in wild-type KRAS cells a complete inhibition of the RAS-RAF-MEK-ERK pathway was observed, whereas in cells with mutated KRAS^G12C^ SOS1 inhibition led to a reduction in ERK phosphorylation by approx. 50% (154). This finding raised the question whether combinatorial inhibition of additional targets could enhance the observed partial inhibitory effect on the RAS-RAF-MEK-ERK signaling cascade. Indeed, vertical dual inhibition with a covalent inhibitor of KRAS^G12C^ resulted in synergistic anti-tumor activity (154). Therefore, SOS1 inhibition has been identified as a viable option and promising tool in therapeutic regimens consisting of various combinatorial therapeutic agents targeting mutant RAS-driven tumors and other tumors with EGFR and NF1 alterations (154, 156). First clinical trials have been initiated investigating the potential of SOS1 inhibition in patients with solid tumor malignancies with KRAS^G12C^ mutation, however with no results reported yet (NCT04111458, NCT04975256).

## RAS-RAF-MEK-ERK hyperactivation and associated immunomodulatory effects

**Figure 2:**
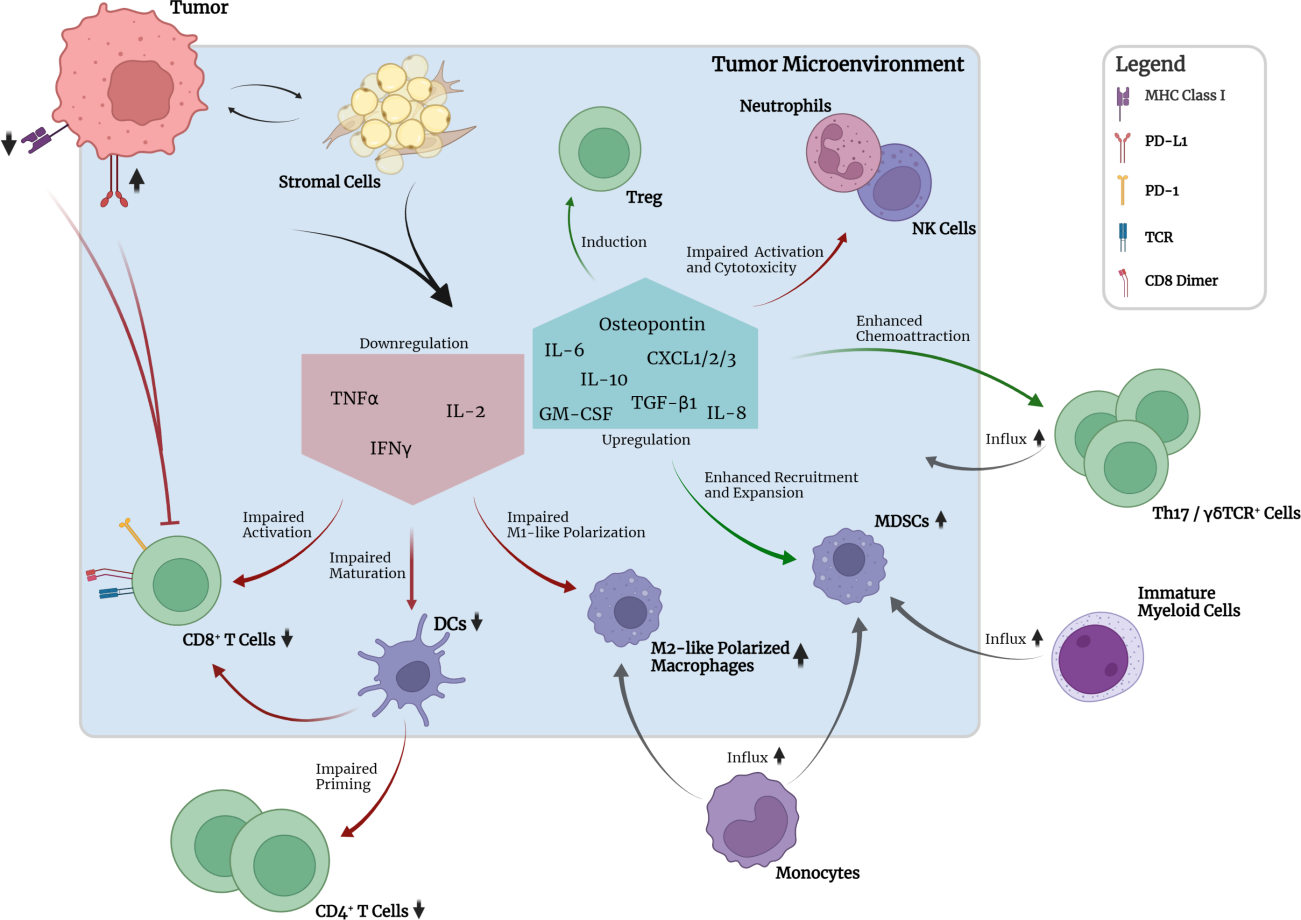
Crosstalk between tumor, TME and selected immune cell types in the absence of pharmacological interruption. Mutant KRAS-associated downregulation of MHC class I molecules and increased expression of PD-L1 on tumor cells leads to reduced detectability and pronounced inhibition of CD8+ T cells. Crosstalk between tumor and stromal cells leads to downregulation of pro-inflammatory cytokine expression, e.g., TNFα, IL-2, and IFNγ, by stromal cells. Consequently, activation of CD8+ T cells, maturation of DCs and M1 polarization of macrophages are impaired. Subsequent priming of CD4+ T cells is impaired. Upregulation of IL-10 and TGF-β1 leads to induction of suppressive peripheral Treg. GM-CSF in the TME together with tumor-secreted osteopontin leads to recruitment and expansion of immunosuppressive myeloid- and monocyte-derived MDSCs. Upregulated expression of chemokines leads to chemoattraction of Th17 and γδTCR+ cells which have been implicated in promotion of tumor cell proliferation and angiogenesis. Neutrophil and NK cell function seem to be impaired regarding activation and cytotoxicity. Upregulation of IL-6 production promotes cell survival and establishment of an inflammatory microenvironment required for optimal tumor growth.

Increasing evidence has shown that oncogenic mutations such as KRAS mutations are capable of mediating crosstalk with the immune system *via* oncogenic signaling and can lead to immune escape mechanisms at different stages of the cancer-immunity cycle (**Figure 2**) (6, 7). During tumorigenesis, the degree of inflammation and inflammatory responses play a key role and concurrently influence the efficacy of various therapeutic approaches. A large variety of tumor-infiltrating immune cells, including CD4^+^ and CD8^+^ T lymphocytes, regulatory T cells (Tregs), B cells, Th17 cells, NK cells, DCs, MDSCs and neutrophils, interact with tumor cells in a complex way, mediated by the TME (**Figure 2**). The TME can be seen as a functional entity made up of the infiltrating immune cell types and stromal cells consisting of fibroblasts, adipocytes, endothelial cells, and the extracellular matrix (ECM) (7, 157, 158). These different cell types interact with each other in a contact- or soluble molecule-dependent manner (autocrine and/or paracrine) and ultimately tilt the balance towards either a pro- or anti-tumorigenic state. Regarding the interaction with the immune system, the two following characteristics of cancer cells have been branded hallmarks of cancer: (i) the ability to evade immunological destruction and (ii) maintenance of tumor-promoting inflammation (159). The advancement of the understanding of KRAS-driven carcinogenesis and its underlying mechanisms has shown that KRAS mutations are not only capable of leading to sustained proliferation and reduced apoptosis in cancer cells but can take direct influence on the TME through autocrine and paracrine effects. Subsequently, the surrounding stromal cells are remodeled by a cytokine-, chemokine- and/or growth-factor driven process in order to establish tumor-promoting inflammation and evasion from the immune system. The resulting immunosuppressive microenvironment prevents tumor antigen (neoantigen) presentation by antigen-presenting cells (APCs) for T cell priming and promotes polarization of macrophages towards the tumor-supportive M2-like phenotype (160–163).

Regarding the establishment of tumor-promoting inflammation, KRAS was shown to be capable of inducing cytokine secretion such as IL-6 and reduced secretion of IFNγ, TNF, and IL-2 by different stromal cell types, e.g., fibroblasts, myoblasts and epithelial cells. This reduced secretion of IFNγ, TNF, and IL-2 was shown to lead to impaired maturation of DCs, CD8^+^ T cell activation and expansion and polarization of M1-like (proinflammatory) macrophages (161). Increased IL-6 secretion was ultimately shown to lead to IL-6-mediated Janus activated kinase (JAK) activation in tumor cells with subsequent downstream phosphorylation and activation of the transcription factor signal transducer and activator of transcription 3 (STAT3) (164). In various studies, this so-called IL-6/JAK/STAT3 axis has been implicated to be the main contributor to several tumorigenic cellular processes, especially in lung and pancreatic cancer, by promoting cell survival and upregulation of the reactive oxygen species (ROS) detoxification program *via* activated MAPK and PI3K/AKT pathways (165–168). Furthermore, IL-6 was shown to be necessary to maintain the activation status of stromal fibroblasts and the inflammatory microenvironment required for optimal tumor growth (168). Besides IL-6, scientific evidence has also pointed towards IL-8, the ligand of CXCR2, to be a critical player in the maintenance of inflammation, tumor growth and also angiogenesis (169). Various studies have uncovered a KRAS/IL-8 link mediated by the MAPK or PI3K/AKT signaling pathways in different human cancer cell lines and tumor specimens (170). Similar to IL-6, IL-8 and CXCR2 signaling was shown to affect cancer-associated fibroblasts and cause increased secretion of pro-tumorigenic cytokines mediated by the nuclear factor kappa-light-chain-enhancer of activated B cells (NF-κB) transcription factor (170). NF-κB itself was also discovered to be a key mediator and inducer of tumor-promoting inflammatory responses (171).

Other more recent scientific efforts have indicated that activating KRAS mutations could lead to the chemoattraction of macrophages and Th17 cells, which have both been implicated in inflammation-induced tumorigenesis by secretion of proinflammatory cytokines, e.g., TNF, and proteases, e.g., matrix metalloprotease-9 (MMP-9) (172–176). Moreover, activation of KRAS was also reported to be associated with increased numbers of γδTCR^+^ inflammatory cells, which are a non-MHC-restricted lymphocyte subset closely associated with innate immunity and implicated in accelerated tumor formation (174). γδTCR^+^ cells were shown to produce high levels of IL-17, leading to promotion of tumor cell proliferation, angiogenesis, production of proinflammatory cytokines and chemokines, metalloproteases, and MDSC attraction (173). Furthermore, γδTCR^+^ cells were found to suppress tumor-infiltrating and anti-tumorigenic αβTCR^+^ cells through checkpoint receptor ligation (177). In line with these findings, genetic or pharmacologic ablation of γδTCR^+^ cells led to protective effects regarding tumorigenesis in a murine model (177). Furthermore, recent studies have identified the inflammasome, a danger-sensing multimeric protein complex part of the innate immune response, to play a crucial role in KRAS-driven leukemia (178). Oncogenic KRAS in leukemia was shown to cause the activation of the NLRP3 inflammasome, which appeared to take on a key role in the development of cytopenia, splenomegaly and myeloproliferation (178). Whether this finding is also relevant in KRAS-driven solid tumors requires further research.

Besides the establishment of a tumor-promoting inflammation in the TME through a wide variety of mechanisms, KRAS itself has also been associated with immunomodulatory and immune escape effects (**Figure 2**). As such, oncogenic KRAS mutations have been linked to programmed cell death 1 ligand 1 (PD-L1) expression in cancer cells of lung or pancreatic origin (179). Tumor cells in for instance lung and pancreatic cancer have been found to be capable of acquiring the ability to (over)express PD-L1 which facilitates immune evasion by reduction of the tumor-specific T cell function and is correlated with poor OS in several types of solid tumors (179, 180). In KRAS-mutant tumors, upregulation of PD-L1 expression was shown to be regulated by MAPK-dependent transcriptional activity of ERK1/2, AP-1 and partially STAT3 (13, 179, 181). However, one study showed that PD-L1 expression was instead regulated by the activation of PI3K/AKT downstream of KRAS (182). Besides upregulating the transcriptional activity of the PD-L1 gene, KRAS-mutant tumor cells were also shown to be able to increase PD-L1 mRNA stability *via* MAPK-dependent signaling (183). Importantly, the associations between KRAS activation and the expression of PD-L1 should always be interpreted in the context of the respective tumor origin, as studies have revealed that KRAS mutations in CRC can result in inverted effects on PD-L1 expression levels compared to lung or pancreatic cancer (184, 185). Turning to other immunomodulating mechanisms, KRAS-mutant tumors also appeared to enhance local immunosuppression by peripheral induction of regulatory T cells (Tregs) in the TME through increased IL-10 and TGF-β1 secretion mediated by the MEK-ERK and AP-1 pathway (186). Another immunomodulatory effect of KRAS-mutant tumors was found to be the downregulation of MHC class I molecules on the cell surface of tumor cells through MAPK-driven internalization and intracellular sequestration. As a result, the recognition of tumor-associated antigens or neoantigens by effector CD8^+^ T cells through MHC class I molecule and TCR interaction was hindered, rendering the tumor cells less recognizable and less sensitive to lysis (7, 10, 161, 187). Furthermore, KRAS mutations were shown to upregulate GM-CSF expression in the TME, enhancing the infiltration of MDSCs which are known to be potent suppressors of effector T cell responses and therefore contribute to the evasion of anti-tumor immunity (187–190). Another mechanism involved in the chemoattraction of MDSCs to the TME was shown to be oncogenic KRAS-induced repression of interferon regulatory factor 2 (IRF2) resulting in higher expression of CXCL3 which binds to CXCR2 on MDSCs and promotes their migration to the TME (191).

Taken together, research efforts of the past have clarified that oncogenic KRAS-signaling is capable of inhibiting or modulating immune responses on various levels in order to dampen or even completely silence anti-tumor immune responses at different stages of the cancer-immunity cycle and enable unchecked tumor growth. This increased understanding has led to the discovery of many new opportunities regarding therapeutic approaches in the ongoing fight against cancer.

## Pharmacological interference with the RAS-RAF-MEK-ERK pathway hyperactivation through MEK inhibition and associated immunomodulatory effects

Since immunomodulatory effects of KRAS-driven oncogenic signaling have been identified to play a major role in tumorigenesis and maintenance. Moreover, MEKi have been shown to be promising and feasible in particularly combinatorial therapy regimens not only regarding direct anti-cancer effects but also anti-tumor immunomodulatory effects. Although MEKi have primarily been developed for inhibition of oncogenic signaling, recent scientific interest in their immunomodulatory effects when administered systemically has substantially risen due to promising results after targeted inhibition of MEK in combination with immunotherapeutic agents in certain tumor types with high immunogenicity (10). As various studies have shown, the involvement of the MAPK pathway in tumorigenesis and immune function, e.g. initiation of innate immunity, activation of adaptive immunity or establishment of the TME, is complex and context-dependent and requires further elaboration regarding MEKi-associated alterations to immune regulation of specific immune cell subsets and the TME (10).

### MEKi and T lymphocytes

MEKi have been shown to influence T lymphocyte physiology and function on various levels. *In vitro* priming of T cells revealed that MEK inhibition blocks priming and expansion of naïve CD8^+^ T cells in response to anti-CD3 and anti-CD28 stimulation (11). In order to identify whether MEK inhibition would also influence priming *in vivo*, tumor-draining lymph nodes were evaluated for conversion of naïve CD8^+^ T cells into fully differentiated cytotoxic T-bet^hi^Eomes^lo^ T cells (11). In line with the *in vitro* findings, MEKi treatment led to reduced numbers of T-bet^hi^Eomes^lo^ T cells in tumor-draining lymph nodes. Also, the number of tumor-antigen-specific T cells in the lymph nodes was reduced. Withdrawal from MEKi treatment restored the MEKi-associated effects, indicating that MEKi treatment does not deplete naïve precursor cells and that its effect on tumor-antigen-specific T cells is reversible (11). In a different murine study, DC function, antigen-induced T cell priming and proliferation upon antigen presentation showed slight reduction at MEKi dosages sufficient for suppression of tumor growth only when co-administered with anti-CD40 agonistic Ab, supporting the notion that CD40 signaling could overcome the impaired priming (192). Other *in vitro* studies have shown that MEKi are capable of suppressing proliferation and cytokine production, e.g., IL-2 through TCR signaling blockade (11, 13, 193, 194). Cell culturing in the presence of trametinib showed partial inhibition of CD4^+^ T cell proliferation after 3 days of treatment. Interestingly, the inhibitory effect vanished after 7 days of cell culturing (161). The negative effects found *in vitro* could however not fully translate to *in vivo* models (10). In a *BRAF^V600E^-*driven melanoma mouse model, the T cell inhibitory effects seen *in vitro* could not be observed *in vivo* (195). T cell lytic activity, infiltration, cytotoxicity and their response to stimulation with subsequent cytokine release (IFNγ) appeared to be normal (195). However, in another study on trametinib in an ovarian cancer mouse model, effector CD8^+^ T cells were shown to proliferate to a lesser extent after treatment with the MEKi. Also, IFNγ production by MEKi-treated CD8^+^ and CD4^+^ T cells isolated from the mouse tumors appeared to be significantly reduced (24). The inconsistent results found by the afore mentioned studies could be explained by differences in treatment schedules and the co-administration of exogenous IL-2 in the utilized functional assays in ref. 153. Interestingly, in a different study, decreased T cell proliferation and IFNγ production in the presence of MEKi was shown to be the result of decreased IL-2 secretion. This effect was shown to be reversible upon exogenous IL-2 administration (10, 23).

When looking closer at tumor-infiltrating CD8^+^ T cells after MEK inhibition, notable differences in the expression of the transcription factors T-bet and Eomes were observed (11). In control tumors without MEK inhibition, the infiltrating CD8^+^ T cell population primarily consisted of the T-bet^lo^Eomes^lo^ phenotype with virtually no T-bet^hi^Eomes^hi^ cells (11). In MEKi-treated tumors, the majority of tumor-infiltrating CD8^+^ T cells (approx. 70%) expressed T-bet. 20 – 25% of the CD8^+^ T cells were double positive for T-bet and Eomes. A closer look at antigen-specificity of these T cells revealed that the MEKi-induced appearance of T-bet^hi^Eomes^lo^ and T-bet^hi^Eomes^hi^ CD8^+^ T cells included the accumulation of tumor-antigen-specific effector T cells that could directly target the tumor cells (11). Upon *in vitro* restimulation, CD8^+^ T cells from the MEKi-treated tumors exhibited more pronounced effector characteristics than T cells from control tumors measured by IFNγ production, supporting the notion of MEK inhibition leading to the presence of a more potent tumor-infiltrating effector T cell population (11). These observations were further substantiated in a recent study on MEKi-induced metabolic reprogramming of effector CD8^+^ T cells (196). MEK inhibition led to the generation of antigen-experienced T memory stem cells (T_SCM_) with a very strong cellular fitness giving rise to highly activated and less exhausted CD8^+^ T cells with high antitumor activity (196). In chronic virus infections and cancer, the occurrence of T cell exhaustion ultimately leading to apoptosis as a consequence to chronic exposure to antigens and inflammatory signals has been frequently observed (11). Typically, inhibitory surface molecules including PD-1, CTLA-4, T cell immunoglobulin and mucin-domain containing 3 (TIM-3), and Lymphocyte activation gene 3 (LAG-3) are significantly upregulated, limiting response to antigen-mediated TCR stimulation, proliferation, and secretion of effector cytokines (197). In exhaustion, the NR4A transcription factor family has been implicated as a master regulator of especially CD8^+^ T cells (197). Interestingly, maximal NR4A activity in response to TCR stimulation has been shown to be dependent on intact ERK signaling, offering a rationale for the reported MEKi-associated effects on T cell exhaustion (196, 198). Although negative feedback cycles, such as activation of the PD-1 receptor, could also slow down or prevent exhaustion in T cells, maintenance of highly active cytotoxic T lymphocyte effectors is critical for cell-mediated anti-tumor responses (11). Prolonged blockade of TCR signaling by means of MEK inhibition was shown to interfere with effector function and proliferation of T cells at the tumor site (24). It was also shown that temporary MEK inhibition would transiently inhibit cell-cycle progression after TCR-mediated cell activation in naïve CD8^+^ T cells, creating a window of opportunity for the generation of functionally and metabolically enhanced CD8^+^ T_SCM_ (196). Later on, after withdrawal of the MEKi and upon restimulation, these CD8^+^ T_SCM_ would then reinstate their proliferative capacities to generate highly potent and robust CD8^+^ T cells, as described above (196).

**Figure 3:**
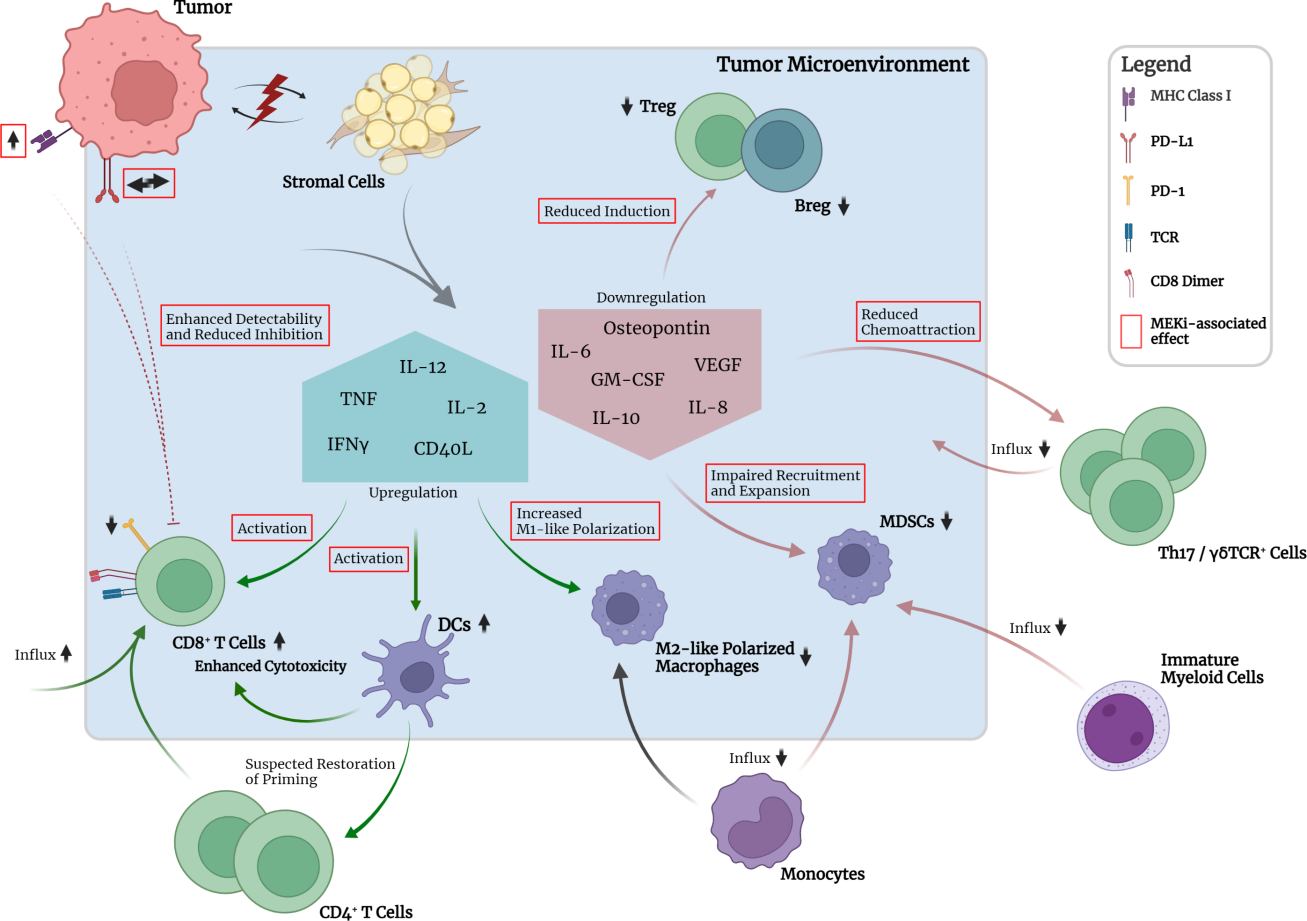
MEKi-associated cell intrinsic effects in selected immune cell types. MEKi leads to transient suppressed proliferation and cytokine production, e.g., IFNγ and IL-2, in CD4+ and CD8+ T cells through prolonged TCR signaling blockade. Infiltration, cytotoxicity and cytokine release upon (re-)stimulation however appear normal. Tumor-infiltrating CD8+ T cells showed MEKi-induced increased expression of the transcription factors T-bet and Eomes leading to accumulation of more potent tumor-antigen-specific CD8+ T effector cells. Transient MEKi-associated cell-cycle progression halt leads to generation of metabolically enhanced T memory stem cells (TSCM), which give rise to potent and robust CD8+ T effector cells upon restimulation. Lower expression of PD-1 was observed in tumor-infiltrating CD8+ T cells, suggesting these cells being less prone to exhaustion and inhibition. MEK inhibition disrupts chronic BCR signaling in Breg resulting in diminished expression of suppressive surface molecules and reduction of Breg numbers removing inhibitory action on CD4+ and CD8+ T cells. MEKi treatment of NK cells does not impair viability, even at high MEKi concentrations. However, proliferation, expression of activation markers and cytolytic capacities are significantly reduced. Cell cycle progression and cellular survival mediated through activated eukaryotic translation initiation factor 4E (eIF4E) is regulated by the MAPK pathway. MEKi disrupts RAS-RAF-MEK-ERK dependent M2 polarization and shifts macrophages towards p38 MAPK dependent M1 polarization. MEKi can restore production of IL-12 and TNF in DCs reversing tumor-induced downregulation of costimulatory molecules and activation status. MEKi-associated reduction of IL-6 expression by tumor and stromal cells restores expression of costimulatory molecules, activation markers, antigen presenting receptor CD1a and functionality. In MDSCs, MEK inhibition with subsequent prevention of ERK phosphorylation led to strongly reduced accumulation in the tumor microenvironment by inhibiting cell expansion and promoting apoptosis. Furthermore, MEKi can abrogate cytokine-induced MDSC expansion.

Taken together, the potential effects of MEK inhibition on T lymphocytes have been investigated extensively (**Figure 3**). Nevertheless, it has become apparent that an unambiguous conclusion cannot be drawn yet due to inconsistent results on priming, proliferation and effector function in various studies. Importantly, there is a need for a standardized approach with regard to experimental design in order to further clarify the true MEKi-associated effects on T lymphocytes.

### MEKi and B lymphocytes

Although the precise role of B lymphocytes in the anti-tumor immune response has received comparably little scientific attention, recent research efforts have been gathering an emerging body of evidence recognizing a role for B cells in modulating the immune response in cancer. Regulatory B cells (Bregs) have been identified to be a heterogeneous cell population capable of suppressing effector T cell function and promoting immune tolerance (10, 199). The BCR is known to activate the downstream positioned RAS-RAF-MEK-ERK pathway in order to induce B cell responses to the recognized antigen, as discussed above (200). MEK inhibition was found to reduce Bregs *in vitro* and *in vivo* in a model of CRC. Although the precise mechanism behind this finding remains unclear, it is proposed that MEK inhibition disrupts chronic BCR signaling resulting in diminished expression of specific suppressive surface molecules and leading to the reduction of Breg numbers (**Figure 3**). In line with the decreased Breg population upon MEK inhibition, numbers of tumor-infiltrating CD8^+^ and CD4^+^ T cells were found to be increased (199).

### MEKi and NK cells

Natural killer (NK) cells together with cytolytic T lymphocytes (CTL) are known to play an important role in cancer immune-surveillance through their potent natural cytotoxic activity. Two distinct mechanisms of cytotoxicity have been identified, i.e. perforin and granzyme-dependent and Fas ligand-dependent (201). Interestingly, it has been proposed in the past that healthy individuals with low overall cytotoxic activity are at a significantly higher risk of developing cancer compared to individuals with medium or high cytotoxic activity (202). Moreover, increased tumor-infiltration by NK cells correlates with a better prognosis in different human tumors (203–205). *In vitro* MEKi treatment of NK cells does not impair viability, even at high MEKi concentrations. However, proliferation, expression of activation markers and cytolytic capacities are significantly reduced (**Figure 3**). IL-15/IL-18 substitution could rescue the cells from the observed detrimental MEKi-associated effects (201). In line with these findings, another study revealed cell cycle progression and cellular survival of NK cells mediated through activated eukaryotic translation initiation factor 4E (eIF4E) to be regulated by the MAPK pathway. Furthermore, it was shown that IL-2 and IL-15 were both capable of activating the MAPK pathway leading to increased eIF4E activity, which could explain the rescue of NK cell functionality in the presence of IL-15 (201, 206). It is of great importance to investigate whether these effects seen in *in vitro* experiments can be reproduced *in vivo* and how these potentially negative effects of MEKi on NK cell expansion and function could be countered.

### MEKi and macrophages

Recent studies investigating the effect of MEKi on macrophage polarization in the TME revealed a shift in the macrophage subset balance upon MEK inhibition from tumor-nurturing M2 macrophages towards the proinflammatory M1 macrophage type. M2 macrophages were shown to critically depend on the intact signaling cascade of the RAS-RAF-MEK-ERK pathway, whereas M1 macrophages rely more heavily on the p38 MAPK signaling pathway rescuing them from cell death under MEK inhibition (**Figure 3**) (9). This finding indicates that MEK inhibition can not only mediate direct antiproliferative and cytotoxic effects on tumor cells but could also shift the balance of the immune setting towards an anti-tumorigenic immune setting by depletion of immunosuppressive macrophages (9, 192).

### MEKi and dendritic cells

It has been proposed that differentiation and function of DCs in cancer patients are impaired due to interaction with tumor cells or tumor-derived cytokines, in particular IL-6. Interestingly, it was shown that the tumor-derived cytokines are able to activate the p38 MAPK pathway and STAT3 signaling in DCs, leading to lower expression levels of costimulatory molecules (i.e., CD40, CD80), activation markers (e.g., HLA-DR) and CD1a, and functionally abnormal DCs (**Figure 3**) (15). These findings are in contrast to the earlier reported maturation-enhancing influence of p38 pathway activity in the maturation process of DCs, possibly pointing at opposite outcomes of p38 activity dependent on maturation status (87). Indeed, *in vitro* neutralization of IL-6 and inhibition of p38 restored the observed abnormalities and function of the DCs (15). In another study, DCs were exposed to *in vivo* MEK inhibition in a mouse model and did not seem to be impaired in their function regarding antigen uptake, processing and presentation to lymphocytes, opening the door to DC-based vaccination as a combinatorial approach in anticancer therapy (14, 192). In particular, Fischetti et al. reported cytokine release in response to TLR ligation to be predominantly dependent on intact p38 and JNK signaling but not ERK signaling (207). Also, MEKi treatment was shown to partially or completely restore the suppressed production of IL-12 and TNF in DCs mediated by melanoma cells and reverse the melanoma-induced downregulation of costimulatory molecules and activation markers of DCs (14).

### MEKi and myeloid-derived suppressor cells

Chronic inflammatory responses and expression of specific chemoattractants such as CXCL1/2/3/8 by tumor cells during tumorigenesis promote the pathologic recruitment and expansion of MDSCs in the TME which contribute to the occurrence of immune evasion from adaptive and non-specific immune responses (12, 208). Normal myeloid cell differentiation is diverted from its intrinsic pathway of terminal differentiation into mature myeloid cells, i.e., DCs, macrophages and granulocytes, towards the generation of pathologically activated MDSCs. Tumor-derived factors have been demonstrated to regulate myeloid cell responses and initiate immunosuppressive pathways committing immature myeloid cells to become MDSCs (12). MEK and ERK signaling have been identified to play an important role in lineage commitment in myeloid cells from hematopoietic stem cells and multipotent progenitor cells (209, 210). In tumor-driven MDSCs, the requirement for RAS-RAF-MEK-ERK signaling in cell expansion has not been studied extensively (211). However, MEK inhibition with subsequent prevention of ERK phosphorylation led to strongly reduced accumulation of MDSCs in mouse tumor models by inhibiting the inflammation-induced cell expansion and promotion of apoptosis (12, 192, 212). Additionally, the secretion of tumor-derived osteopontin, a chemoattractant for MDSCs, was reduced after MEKi treatment correlating with a decrease in MDSCs (12). Furthermore, *in vitro* studies showed that MEK inhibition is capable of abrogating cytokine-induced MDSC expansion (12). It has been proposed that specifically IL-6 is capable of promoting MDSC expansion and also significantly improves the immunosuppressive capacity of MDSCs (208). Subsequent RNA sequencing and proteomic analyses in MDSCs treated with IL-6 revealed that especially the RAS-RAF-MEK-ERK signaling cascade was upregulated, suggesting a relationship between IL-6, cell expansion and enhanced immunosuppression (**Figure 3**) (208). These findings suggest that MEK inhibition does not only contribute to creating MDSC-hostile conditions in the TME by attenuating the expression of immunosuppressive factors by tumor and stromal cells but might also directly influence intracellular, RAS-RAF-MEK-ERK-dependent proliferative and prosurvival processes in MDSCs, leading to an overall increased protective anti-tumor immune response (12, 208).

### MEKi and tumor cell-TME crosstalk

**Figure 4:**
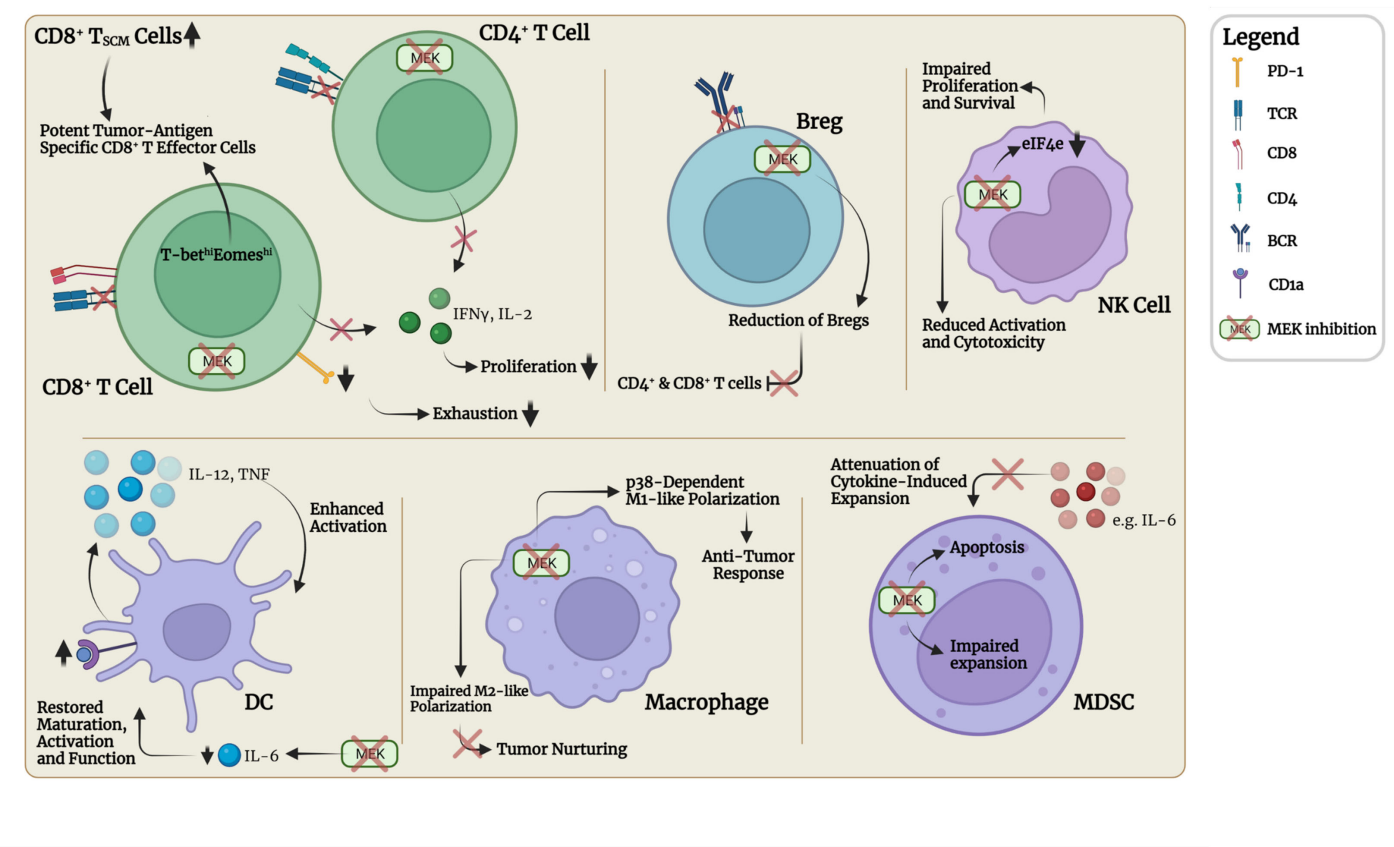
MEK inhibition and associated immunomodulatory effects in the TME (marked with red boxes). MEKi interrupts immune evasion mechanisms and tumor crosstalk with stromal cells and restores an anti-tumorigenic microenvironment by increasing the expression and/or release of proinflammatory molecules, e.g., CD40L, IFNγ, TNF, and CD40L, while simultaneously reducing the presence of immunosuppressive cytokines, e.g., IL-6, IL-10, GM-CSF, and IL-6, and suppressive cell populations such as Tregs, Bregs, MDSCs and M2-polarized macrophages. MEKi also induces upregulation of MHC class I molecules, thereby increasing detectability of tumor cells by CD8+ T effector cells and leading to enhanced infiltration. PD-L1 expression by tumor cells remains either unchanged or variable. Loss of VEGF expression by tumor cells could potentially improve CD8+ T cell infiltration through reduction of abnormal formations of tumor vessels. Furthermore, DC activation and expression of costimulatory molecules is restored leading to enhanced priming and activation of CD4+ and CD8+ T cells.

Regarding the capability of KRAS and BRAF mutant tumors to create an immunosuppressive microenvironment by interfering with the cancer-immunity cycle, various studies have shown that MEKi can restore an immune stimulatory and anti-tumorigenic microenvironment. This effect was achieved by increasing the expression and/or release of proinflammatory molecules, e.g. CD40L or IFNγ, while reducing the presence of immunosuppressive cytokines, e.g. IL-6 and IL-10, and suppressive immune cells such as Tregs, MDSCs, and tumor-associated macrophages (TAMs) (**Figure 4**) (161). KRAS activation in non-hematopoietic microenvironment caused malignant transformation of hematopoietic cells (213). Gene expression analysis of sorted nonhematopoietic BM niche cells from *Kras^G12D^* mice revealed upregulation of multiple inflammation-related genes including IL1-superfamily members (*Il1α, Il1β, Il1f9*) and the NLPR3 inflammasome (213). The immunosuppressive cytokines were shown to promote the accumulation of immunosuppressive cells leading to inhibition of anti-tumor immunity through various mechanisms, e.g. depletion of arginine necessary for T cell growth, recruitment of Tregs and increase in (ROS) (13). Utilization of MEKi was shown to result in decreased mRNA and protein levels of such immunosuppressive cytokines and subsequent restoration of a proinflammatory environment with increased activity of anti-tumor immunity (13). Moreover, MEKi were shown to hinder the recruitment of monocytic MDSCs to the TME and inhibit monocyte differentiation into TAM. MDSCs and TAM are known to be heterogeneous and plastic cell populations capable of hindering anti-tumor immunity by blocking effector T cell functioning through iNOS and arginase production, which would respectively lead to increased ROS production and arginine depletion (12). Furthermore, these cell populations have been implicated in the direct promotion of tumor growth through stimulation of angiogenesis and metastasis formation (12). MEKi treatment could therefore slow these processes from occurring and support the restoration of an adequate anti-tumor immune response (10).

Next, application of different MEKi induced the upregulation of MHC class I molecules in various tumor models, suggesting increased detectability of tumor cells by effector CD8^+^ T cells (**Figure 4**) (161). In line with this finding, a pooled human kinome shRNA interference-based approach led to the identification of MEK to be a negative regulator of MHC class I expression (214). Furthermore, several studies linked MEKi-associated CD8^+^ T cell infiltration of tumor tissue with the loss of vascular endothelial growth factor (VEGF) expression by tumor cells (161, 215). Abnormal formations of tumor vessels due to hyperexpression of VEGF have been hypothesized to impede the influx and subsequent penetration of tumor tissue by immune cells (216).

Regarding the important role of PD-L1 expression by tumor cells, as described above, several studies have seen no or variable changes in PD-L1 expression in various cancer cell lines upon administration of MEKi, dependent on specific cell line, experimental context or duration of MEK inhibition (13, 183). Interestingly, analysis of PD-1 expression on tumor-infiltrating CD8^+^ T cells revealed that in MEKi-treated tumors almost 40% of the infiltrating CD8^+^ T cells were PD-1^lo^, whereas in control tumors 85% of infiltrating CD8^+^ T cells expressed high levels of PD-1 (11). A precise understanding of these unexpected and in some cases contradictory observations is still lacking, although escape mechanisms through acquisition of molecular resistance pathways have been proposed. A better understanding of the genetic and proteomic background on tumor PD-L1 regulation is required (13). Besides investigating the effect of PD-L1/PD-1 interruption, inhibition of other immune checkpoints including cytotoxic CTLA-4 and TIM-3 in the presence of MEK inhibition has also come into the focus of recent research efforts (27, 217, 218).

## Potential advantages of combinatorial targeted therapy and intermittent administration of selective inhibitors

RAS, MEK or ERK kinase inhibitors have the potential to cause significant toxicities at higher dosages. Furthermore, various resistance mechanisms against these kinase inhibitors have been shown to lead to transient anti-tumor effects. Therefore, therapy regimens utilizing combinations of different selective inhibitors targeting both upstream and downstream proteins or parallel pathways have received continuously growing scientific attention in an attempt to overcome these hurdles (32). For example, the combination of MEK or KRAS^G12C^ inhibitors and SHP2 or SOS1 inhibitors has led to promising results in preclinical studies for the treatment of KRAS-driven tumors (151, 154, 219). Various clinical trials are currently active, investigating the clinical effectiveness of these therapeutic strategies (**Table 2**) (33). Also, combinatorial approaches utilizing RAF, MEK and/or ERK inhibitors and autophagy inhibitors, which are thought to increase antigen presentation for enhanced immune responses, are being investigated in clinical trials (e.g., NCT04214418, **Table 2**) (220–222). Particularly simultaneous inhibition of two elements of the RAF-MEK-ERK cascade, also known as vertical inhibition, was proven to result in highly synergistic and apoptotic activity in murine tumor cells that could not be replicated with the administration of only one single inhibitor, even at high dosage regimens (223). Concurrent inhibition of RAF and ERK was shown to be most effective with induction of tumor regression even at low doses without causing significant toxicity (223). In line with this finding, another research group identified the so-called multiple low dose (MLD) therapy with RAF (RAFi) and ERKi as part of either a triple or quadruple vertical combination therapy to be effective in a lung cancer mouse model leading to significant tumor regression without causing major toxicities or acquired resistance in specific patient subsets (224).

In addition to finding the most effective approach in RAS-RAF-MEK-ERK interference, the increased understanding of the impact of tumor-driven immune modulation and crosstalk with the TME has led to extensive investigational work on therapy regimens combining the immunomodulatory effects of MEKi with another selective inhibitor and an immunotherapeutic agent targeting immune checkpoint molecules (ICB), e.g., CTLA-4, PD-1 or PD-L1, or addressing immunostimulatory receptors, e.g., CD40, CD134 or CD137. These efforts have led to the identification of treatment modalities with significant clinical efficacy in specific patient subsets (9, 10, 27). For instance, the interruption of the PD-L1/PD-1 signaling axis in combination with MEKi and BRAFi was shown to have the potential to increase overall response rates and reduce the rate of MEKi/BRAFi-associated resistance in preclinical models (225). In this regimen, comparably lower but longer response rates to PD-L1/PD-1 blockade and higher but shorter response rates to MEKi/BRAFi treatment complement each other for the most optimal therapy efficacy (26, 226–228). Multiple other advantages linked to anti-PD-L1/PD-1 treatment have been reported, including (i) previous immune checkpoint inhibition and lead-in therapy with two doses of anti-PD-L1/PD-1 enhancing subsequent MAPK pathway inhibition and anti-tumor efficacy and (ii) increased tumor-infiltration by iNOS^+^ M1-like tumor-associated macrophages (TAMs), Th1-like Tbet^hi^ CD4^+^ T cells and granzyme B^hi^ CD8^+^ cytotoxic T cells after sequential-combinatorial therapy with improved durability of tumor regression (225). Another interesting approach has been identified to be the concurrent administration of MEKi and ICB together with chemotherapy, which was very recently shown to result in synergistic effects regarding the TME-associated cytokine profile, CD8^+^ T cell recruitment and sensitization to ICB treatment (229).

With immunostimulatory targeting, the administration of for instance an agonistic anti-CD40 Ab alongside a MEKi in a murine model led to the discovery of a potent synergistic impact on MEKi-induced immunomodulation through a dual CD40-mitigated mechanism of action, i.e., enhancement of T cell immunity and modulation of the macrophage TME infiltrate towards a higher M1/M2 ratio by activating the p38 MAPK signaling cascade (9). In line with these findings, a previous murine study by Baumann et al. also demonstrated potent synergy between the investigated MEKi and anti-CD40 treatment with significant tumor growth suppression (192). Mechanistically, the authors showed the observed effects to be T cell-dependent and noticed an increased CD8^+^ T/CD4^+^ Treg ratio after MEKi and anti-CD40 Ab treatment. Furthermore, the authors demonstrated a similar effect of MEKi/CD40 Ab treatment on the M1/M2 ratio in the TME tilting the balance towards the anti-tumorigenic M1 subtype (9, 192). Other promising immunostimulatory targets have been identified to be the two members of the tumor necrosis factor receptor (TNFR) superfamily CD134 (also known as OX40) and CD137. CD137 signaling has been implicated in the activation of CD8^+^ T cells, while CD134 activity was shown to be a driver of T cell-mediated anti-tumor immunity, both having the potential to significantly improve the anti-tumorigenic effect of combinatorial targeted therapy (162). However, an important caveat in combinatorial approaches utilizing sequential ERK pathway inhibition and immunotherapy has been identified to be the risk of development of cross-resistance to immunotherapeutics. After development of BRAFi/MEKi/ERKi resistance, the development of cross-resistance led to the acquisition of a strongly immune-evasive state determined by an remarkably immunosuppressive TME (147). Within the TME associated with cross-resistance, a pivotal role was attributed to CD103^+^ DCs which were identified to be reduced and functionally impaired in cross-resistant tumors (147). Importantly, restoration of DC functionality was shown to be sufficient to restore immunotherapy responsiveness (147). These findings underpin the importance of understanding the process of cancer cell evolution and TME modification during and after targeted therapy and/or immunotherapy. Uniting this improved understanding with data and reports from most recent clinical trials investigating various dual and triple therapy regimens combining MEK inhibition with other inhibitory agents (**Table 1**) will profoundly aid in the ongoing search for effective combinatorial therapeutic strategies.

Besides improving combinatorial therapy regimens, the precise timing and duration of drug administration has also come into focus due to reports of improved efficacy, reduced odds of resistance induction and reduced toxicity, especially with intermittent administration of MEKi either as monotherapy or in combination with other therapeutic agents (215, 230). Furthermore, the continuous administration of MEKi has been reported to potentially interfere with intracellular cell signaling and physiology in immune cells, as described above. The potential benefits of intermittent administration of cobimetinib in combination with the PI3K inhibitor pictilisib were investigated in murine KRAS- and BRAF-driven tumors (106). This approach led to synergistic cell growth inhibition and increased apoptosis. The intermittent dosing of both compounds led to transient pathway knockdown with a positive correlation between dosage and tumor inhibition/regression (106). These findings were further substantiated by another murine study investigating intermittent administration of selumetinib or trametinib (215). However, contrary to expectations based on preclinical studies, a phase I/II clinical trial investigating continuous as well as intermittent combinatorial administration of cobimetinib and pictilisib in patients diagnosed with CRC, NSCLC or PDAC, reported low tolerance and insignificant anti-tumor activity (231). Similarly, a phase II clinical trial in patients with advanced melanoma showed that intermittent administration of cobimetinib with vemurafenib did not result in statistically significant OS benefit compared to continuous administration. Toxicity was only slightly reduced in the intermittent dosage group (232). Two other study groups investigated intermittent dosing trametinib in combination with either afuresertib or lapatinib in patients with various solid tumors and melanoma in phase I/II clinical trials (233, 234). The dosage tolerability of trametinib and afuresertib was well below the recommended dosage due to significant toxicities. A clinically meaningful dosing schedule could not be achieved (234). Huijberts et al. investigated the combination of trametinib with lapatinib and found that toxicities were manageable at approx. 50% of their single agent dosages. The intermittent dosing regimen seemed more tolerable regarding toxicities. Interestingly, histologically confirmed suppression of MAPK pathway activity did not correlate with clinical activity in CRC, suggesting underlying resistance or escape mechanisms dependent on tumor entity (233). A similar observation was made by Van Brummelen et al. who conducted a phase I trial investigating intermittent administration of selumetinib in combination with afatinib in patients with CRC, NSCLC and PDAC. The majority of enrolled patients were diagnosed with CRC (73%). Although confirmed target engagement was observed, a clinical correlation could not be made, possibly due to disease-associated factors of CRC rendering the therapeutic agents less effective (235). Also in patients with BRAF-mutant malignant melanoma, no advantage of the intermittent administration of trametinib with dabrafenib could be found (236). Based on the findings of clinical trials so far, the potential benefit of intermittent MEKi administration remains unclear. Nevertheless, a currently active phase I/II clinical trial (NCT03581487) is seeking to clarify whether intermittent MEKi treatment combined with ICB therapy could lead to significant benefits (27).

Besides investigating therapeutic agents with single target engagement, a treatment regimen with the novel MEK-pan-RAF inhibitor R05126766 has also come into focus regarding intermittent administration. Guo et al. investigated intermittent dosing schedules and anti-tumor activity in a phase I clinical trial in patients with RAS/RAF-mutant solid tumors and multiple myeloma (237). The investigated inhibitor appeared to be tolerable in intermittent dosing schedules with anti-tumor activity in a large fraction of the patients across various tumor types. Therefore, the authors concluded that the inhibitor R05126766 could be considered in a single-agent regimen (237). This finding is in line with another study on R05126766 with similar findings, warranting further evaluation (238).

The transition from the promising preclinical setting towards the human setting with regard to intermittent MEKi either as monotherapy or in combination with various other agents has proven to be challenging with so far discouraging results. More studies are required to further evaluate the combination of multiple selective inhibitors with or without immunotherapeutic agents and further elucidate the precise role of the immune system in MEKi-associated tumor reduction. Moreover, more studies are required regarding different MEKi agents, as their potency, target specificity and half-life (T_1/2_) can vary and lead to different effects on the immune system or tumor response (215). Furthermore, tumor entity-associated factors potentially dictating treatment response need to be further evaluated in order to improve prediction of target response and identify individualized therapeutic approaches as the shortcomings of the “one-size fits all” conviction have been demonstrated more than once.

## Concluding remarks

Urged by the global need for improved therapeutic strategies in the treatment of cancer, scientific efforts have accumulated an increasing body of knowledge on tumor-associated molecular biology, the tumor microenvironment and the involvement of the immune system in various tumor entities. In this context, the precise role of the oncogenic RAS-RAF-MEK-ERK signaling cascade in the establishment and maintenance of a tumor-sustaining and immune evasive microenvironment has been and is still being elucidated as its complexity has not yet been fully comprehended. The increasing knowledge has led to the discovery of various selective and immunomodulatory therapeutic approaches in an attempt to disrupt pathologically hyperactivated RAS-RAF-MEK-ERK signaling and tilt the balance of the tumor-TME-immune system crosstalk towards an anti-tumorigenic setting. Tremendous efforts have been and are being undertaken to achieve a successful transition of selective anti-cancer treatment modalities, particularly MEK inhibition and disruption of the PD-L1/PD-1 axis, from the promising preclinical stage to clinical trials and approval by medical authorities. However, important limitations hampering the clinical transition have been identified concerning limited effectiveness, significant treatment-related toxicities and occurrence of various resistance mechanisms – possibly dependent on disease entities – rendering therapeutic agents less effective than aspired. The challenge of the most recent scientific efforts has therefore been overcoming these limitations while identifying the most effective therapy regimens combining selective inhibitory targeting, immunomodulatory effects of particularly MEKi and ICB or immunostimulatory agents and also chemotherapy. Besides combining different agents in treatment strategies, a more recent step towards treatment optimization has been the development of intermittent dosing schedules or sequential drug administration with pre-specified lead-in periods. Multiple clinical trials investigating these approaches are currently active and incoming results are highly anticipated. Fortunately, the entirety of the ongoing scientific efforts has led to a slow but steady progress and has continued to successfully bring forward new and promising therapeutic agents, e.g., the most recently approved agent Sotorasib, despite significant challenges. Therefore, it is our conviction based on the recent developments that interventions interfering with the hyperactivation of the RAS-RAF-MEK-ERK pathway still carry great potential in the treatment of associated tumor entities, giving cause for new hope in affected patients.

## Author contributions

TA developed the first draft of the manuscript. TA and DAR contributed to the planning, organization, and writing of the manuscript. All authors reviewed the manuscript and provided critical edits. The final version of the manuscript was approved by all authors.

## Funding

This study was supported by the Deutsche Forschungsgemeinschaft, Germany, SFB-1479 – Project ID: 441891347 (to NK, RZ, TB, and DR). TB is further supported by the DFG (Heisenberg professorship, BR 3662/5–1 and BR3662/4–1), the German Cancer Consortium DKTK (projects SORATRAM, NextGen LOGGIC and MTB TAILOR) and the Ministry for Science, Research and Arts of the State of Baden-Wuerttemberg, project BW-VAPO. DR is supported by the German Cancer Aid (grant number 70113697).

## Acknowledgments

We would like to thankfully acknowledge support by the Open Access Publication Fund of the University of Freiburg, Germany.

All figures were created with BioRender.com. The illustrations in the figures do not claim to be exhaustive. The involved cell types, cytokines, and interactions depicted in the figures are exemplary.

## Conflict of interest

The authors declare that the research was conducted in the absence of any commercial or financial relationships that could be construed as a potential conflict of interest.

## Publisher’s note

All claims expressed in this article are solely those of the authors and do not necessarily represent those of their affiliated organizations, or those of the publisher, the editors and the reviewers. Any product that may be evaluated in this article, or claim that may be made by its manufacturer, is not guaranteed or endorsed by the publisher.
